# Craniofacial abnormality with skeletal dysplasia in mice lacking chondroitin sulfate *N*-acetylgalactosaminyltransferase-1

**DOI:** 10.1038/s41598-018-35412-5

**Published:** 2018-11-20

**Authors:** Hiroko Ida-Yonemochi, Wataru Morita, Nobuo Sugiura, Ryosuke Kawakami, Yuki Morioka, Yuka Takeuchi, Toshiya Sato, Shunichi Shibata, Hideto Watanabe, Takeshi Imamura, Michihiro Igarashi, Hayato Ohshima, Kosei Takeuchi

**Affiliations:** 1 Division of Anatomy and Cell Biology of the Hard Tissue, Department of Tissue Regeneration and Reconstruction, Niigata University Graduate School of Medical and Dental Sciences, Niigata, 951-8514 Japan; 2 Department of Oral Functional Anatomy, Faculty of Dental Medicine, Hokkaido University, Sapporo, 060-8586 Japan; 3 Institute for Molecular Science of Medicine, School of Medicine, Aichi Medical University, Nagakute, 480-1195 Japan; 4 Department of Molecular Medicine for Pathogenesis, Graduate School of Medicine, Ehime University, Ehime, 791-0295 Japan; 5 Department of Medical Cell Biology, School of Medicine, Aichi Medical University, Nagakute, Aichi 480-1195 Japan; 6 Department of Laboratory Animal Science, Kitasato University School of Medicine, Sagamihara, 252-0374 Japan; 7 Department of Maxillofacial Anatomy, Graduate School of Medical and Dental Sciences, Tokyo Medical and Dental University, Tokyo, 113-8549 Japan; 8 Department of Neurochemistry and Molecular Cell Biology, Niigata University Graduate School of Medical and Dental Sciences, Niigata, 951-8510 Japan; 9 Trans-disciplinary Research Program, Niigata University, Niigata, 950-2181 Japan; 10 Medical Research Creation Center, Aichi Medical University, Nagakute, Aichi 480-1195 Japan

## Abstract

Chondroitin sulfate (CS) proteoglycan is a major component of the extracellular matrix and plays an important part in organogenesis. To elucidate the roles of CS for craniofacial development, we analyzed the craniofacial morphology in CS *N*-acetylgalactosaminyltransferase-1 (T1) gene knockout (KO) mice. T1KO mice showed the impaired intramembranous ossification in the skull, and the final skull shape of adult mice included a shorter face, higher and broader calvaria. Some of T1KO mice exhibited severe facial developmental defect, such as eye defects and cleft lip and palate, causing embryonic lethality. At the postnatal stages, T1KO mice with severely reduced CS amounts showed malocclusion, general skeletal dysplasia and skin hyperextension, closely resembling Ehlers-Danlos syndrome-like connective tissue disorders. The production of collagen type 1 was significantly downregulated in T1KO mice, and the deposition of CS-binding molecules, Wnt3a, was decreased with CS in extracellular matrices. The collagen fibers were irregular and aggregated, and connective tissues were dysorganized in the skin and calvaria of T1KO mice. These results suggest that CS regulates the shape of the craniofacial skeleton by modulating connective tissue organization and that the remarkable reduction of CS induces hypoplasia of intramembranous ossification and cartilage anomaly, resulting in skeletal dysplasia.

## Introduction

Craniofacial skeletal development is a complex series of events composed of two distinct ossification modes: intramembranous ossification and endochondral ossification. The calvaria, some of facial bone and mandible are formed by intramembranous ossification, and the morphogenesis of which depends upon the balance between proliferation and differentiation of osteoblast-lineage cells. Hence, the cranial base is formed through endochondral ossification, and the growth of synchondrosis determines anterior-posterior cranial base elongation^1^. Therefore, the determination of skull shape involves multiple factors including the timing of ossification in the sutures and the growth of cranial base synchondroses. During craniofacial morphogenesis, proteoglycans play an important role in the organization of extracellular environment^2–5^. In particular, chondroitin sulfate proteoglycan (CSPG) regulates cartilage elements including the nasal wall, mandibular condyle and synchondrosis^6,7^. Chondroitin sulfate (CS) is one of the major glycosaminoglycans and is composed of *N*-acetylgalactosamine (GalNAc) and glucuronic acid (GluUA). CS is widely distributed on the cell surface and in the extracellular matrices of many tissues, especially the brain and cartilage, and has been known to regulate cell morphogenesis by interacting with signaling molecules such as Wnts, growth factors and cytokines in organogenesis^8–11^. Some CSPG such as versican expresses in the cranial sutures and palatal mesenchyme during intramembranous ossification^12,13^. Versican is abundant in the woven bone, and osteoblasts express versican mRNA prominently during mandibular bone development^14^. Furthermore, CS-related signaling molecules, such as Wnts, hedgehog and fibroblast growth factors (FGFs), regulate palatogenesis^15,16^, cranial base development^17^ and cranial suture morphogenesis^18,19^, and these mutant mice exhibited skull deformation including cleft lip and palate^20^. Therefore, it is expected that CS chains and its related signaling molecules determine craniofacial skeletal shape by regulating not only chondrogenesis but also intramembranous ossification.

Chondroitin sulfate *N*-acetylgalactosaminyltransferase-1 (T1) is a key glycosyltransferase in CS biosynthesis and exhibits GalNAc transfer activity in both the initiation and elongation processes^21–27^. To understand the physiological roles of CS and the enzymes involved in CS biosynthesis, we generated CSGalNAcT1 gene knockout (T1KO) mice, which had approximately half the abundance of CS when compared to wild-type mice^28–30^. The epiphyseal cartilage of T1KO mice was significantly smaller than that of wild-type mice, and type II collagen fibers in developing cartilage became abnormally aggregated and disarranged in T1KO limbs^28^. In addition, other researchers have established T1KO mice^31^ or T1 and CSGalNAcT2 (T2) double KO mice^32^ and reported that T1 is required for aggrecan metabolism in cartilage and aberrant ECM caused by CS reduction disrupted endochondral ossification. However, there is little information about the importance of CS and T1 in intramembranous ossification. Therefore, it is well worth analyzing the role of CS and associated signaling molecules in membrane bone formation to understand craniofacial development.

In this paper, we examined the craniofacial morphology of T1KO mice, with a focus on membrane bone. We demonstrated that intramembranous ossification and cartilage formation were disturbed in the cranium of T1KO mice, resulting in the deformation of the craniofacial skeleton. A reduced amount of collagen type 1 and disarranged collagen fibers in the connective tissues were also observed in T1KO mice. These results suggest that CS and T1 play important roles in connective tissue organization and following intramembranous ossification of the craniofacial skeleton, and it is conceivable that a marked CS reduction may cause connective tissue disorders in T1KO mice.

## Results

### Distribution of chondroitin sulfate during craniofacial development

CS chains were widely distributed to the murine craniofacial areas during development (Supplementary Fig. S1). On the embryonic day 18.5 (E18.5), CS was definitely immunolocalized in the mesenchymal cells surrounding newly formed bones in the calvaria and palatal regions, and in the cornea. Versican showed the expression patterns similar to those of CS chains.

### Impaired intramembranous ossification in the neonatal stage of T1KO mice

**Figure 1:**
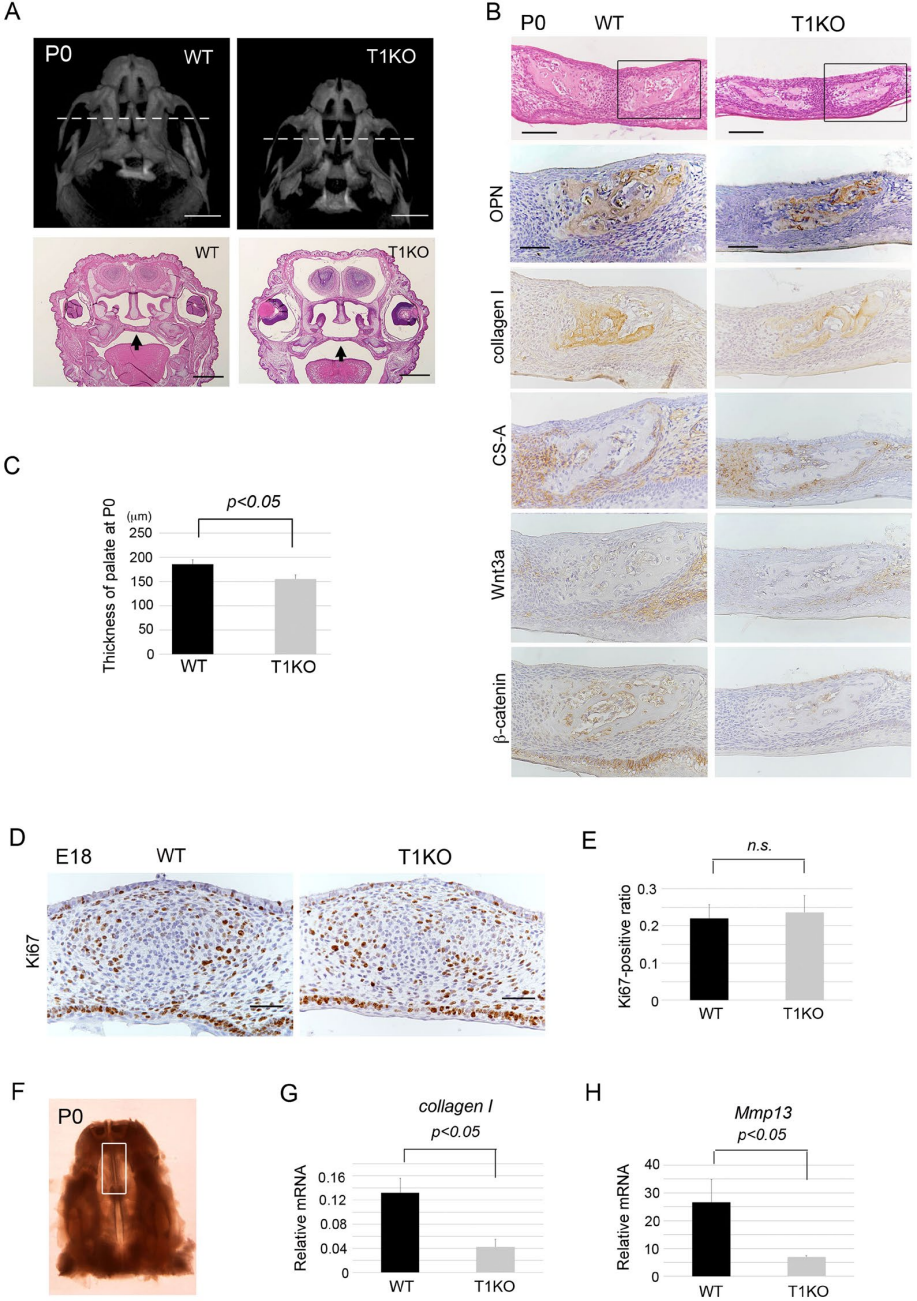
Hypoplastic ossification in the palate at neonatal stages in T1KO mice. Micro CT images on P0, ventral view (**A**). Histological and gene expression analyses of palate of E18 and P0 mice (**B**–**H**). HE and immunoperoxidase staining for osteopontin (OPN), collagen type 1, CS-A, Wnt3a, β-catenin (**B**) and Ki67 (**D**) in the serial section, counter-stained with hematoxylin. (**A**) The ossification of palate was delayed in T1KO pups. To compare palatal formation, the frontal plane crossing the center of bilateral maxillary first molars and eyes (dot lines) was selected. Connective tissue surrounding osteopontin (OPN) and collagen type 1-positive neonatal bone was thinning in T1KO mice, and immunostaining intensity of CS, Wnt3a and β-catenin was attenuated in the palatal mesenchyme of T1KO mice (**B**). Double immunostaining for CS, collagen 1, Wnt3a and β-catenin with OPN were shown in Supplementary Figure S2. The thickness of palate was significantly thin in T1KO on P0 (n = 10 for each, Student’s *t-*test) (**C**). The cell proliferation rate of mesenchymal cells in the palatal regions was not significantly different between WT and T1KO on E18 (**D**,**E**). mRNA expression of *collagen type 1* and *Mmp13* was significantly downregulated in T1KO palate (**G**,**H**). n = 3, Student’s *t* test (**E**,**G**,**H**). Scale bars, 2 mm (**A**, upper); 1 mm (**A**, lower); 100 μm (**B**, upper); 50 μm (**B**, lower); 50 μm (**D**).

**Figure 2:**
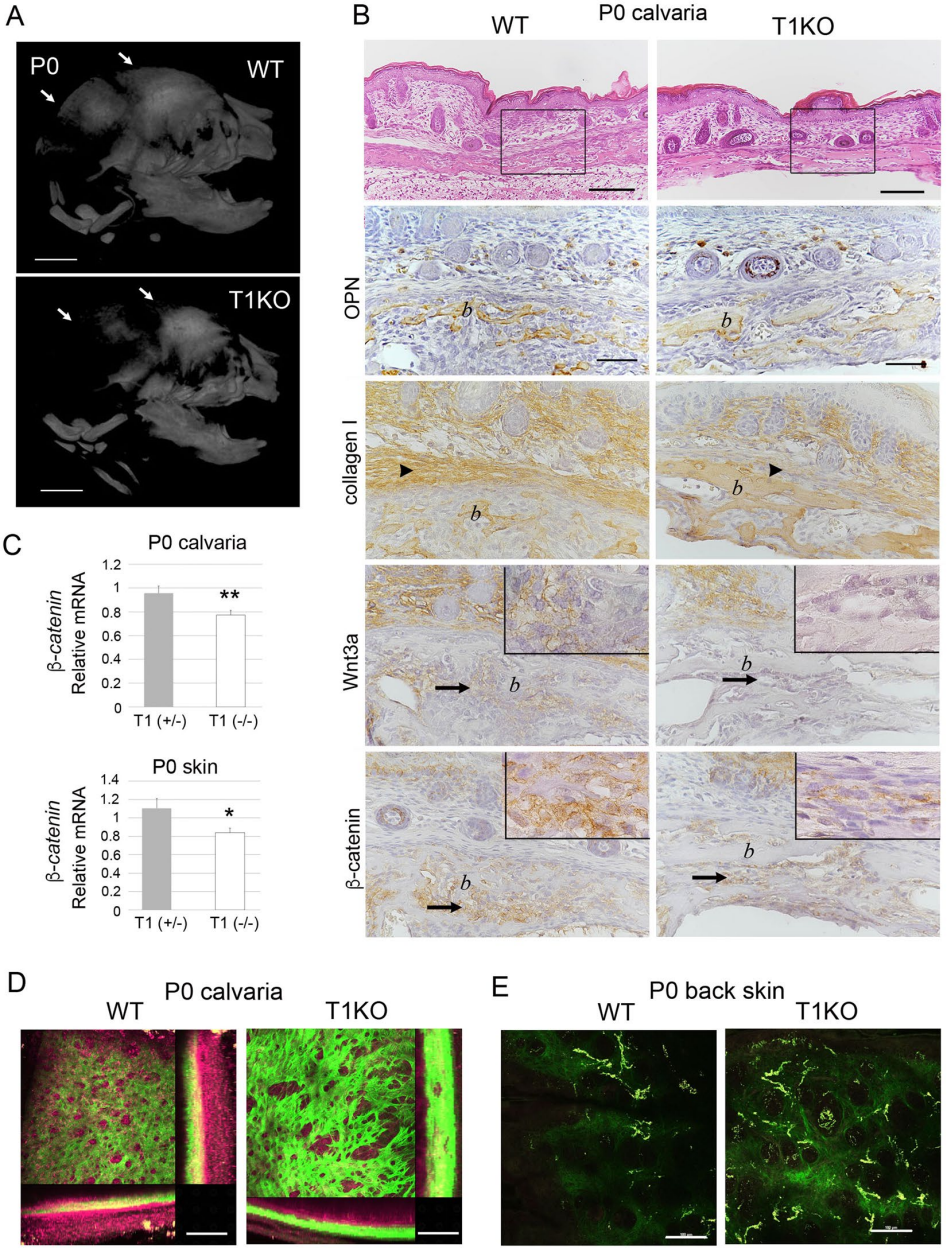
Calvarial formation of WT and T1KO mice at neonatal stage. Micro CT image (**A**), histological and gene expression analyses of calvarial region in P0 mice (**B**,**C**). HE and immunoperoxidase staining for OPN, collagen type 1, Wnt3a and β-catenin, counter-stained with hematoxylin (**B**). (**A**) The ossification of calvarial bones was delayed in T1KO pups (*arrows*). Collagen type 1-positive connective tissues covering bone surface were thin in T1KO mice (*arrowheads*), and the immunoreactivity of Wnt3a and β-catenin in the mesenchymal tissues, surrounding the OPN-positive neonatal bones, was weak in T1KO mice (*arrows*) (**B**). Double immunofluorescent staining for collagen 1, Wnt3a and β-catenin with OPN were shown in Supplementary Figure S4. (**C**) mRNA expression of *β-catenin* in the periosteum and dermis of calvarial region. The average expression of *β-catenin* in WT mice was defined as 1.0, and gene levels were compared with that of WT mice. mRNA expression of *β-catenin* was significantly downregulated in T1 (-/-) (T1KO) pups (**C**). n = 5, Student’s *t-*test. *p < *0*.*05*, **p < *0*.*01*. (**D**,**E**) Label-free multi-photon imaging of calvaria and scalp. Collagen fibers visualized by SHG in *green* were irregular and aggregated in the calvaria (**D**) and the dermis of scalp (**E**) of T1KO mice on P0. Scale bars, 2 mm (**A**); 100 μm (**B**: *upper*, **D**), 50 μm (**B**: *lower*). *b*: bone tissue.

Heads of wild-type (WT) and T1KO mice on postnatal day 0 (P0) and day 28 (P28) were evaluated using micro–computed tomography (micro CT), soft X ray and serial histological sections. On P0, ossification of craniofacial region including palate, calvaria and cranial base was delayed in T1KO pups, as indicated by arrows (Figs 1A and 2A). To observe the early intramembranous ossification of cranial bones, we examined the histological features of the head region in neonates. To compare palatal formation, we selected the frontal plane crossing the center of bilateral maxillary first molars and eyes as indicated in the dot lines of Fig. 1A. The thickness of palate was significantly thinner in T1KO mice than that in WT mice on P0 (WT: 164.5 μm–244.7 μm, mean = 185.4 μm; T1KO: 99. 7 μm–191.5 μm, mean = 154.8 μm, n = 10 for each) (Fig. 1B,C). Connective tissue surrounding osteopontin (OPN) and collagen type 1-positive neonatal bones was thinning in T1KO mice, and immunostaining intensity of CS, Wnt3a and β-catenin was attenuated in the palatal mesenchyme in the osteogenic front of T1KO mice on P0 (Fig. 1B, Supplementary Fig. S2). However, the cell proliferation rates of mesenchymal cells in the palatal region showed no significant difference between WT and T1KO on E18 (Fig. 1D,E). To confirm the connective tissue remodeling in the palatal region, we dissected the palatal tissue under stereomicroscopy as shown in Fig. 1F, and examined the expression of *collagen type 1* and its degrading enzyme, *Mmp13*. The mRNA expression levels of *collagen type 1* and *Mmp13* were significantly downregulated in the T1KO palate (Fig. 1G,H), although the mRNA expression of *Wnt3a* and *Fgf2*, which interact with CS chains, was not significantly decreased in T1KO mice (Supplementary Fig. S3). In the calvarial region on P0 (Fig. 2), the ossification of calvarial bones was delayed in T1KO pups (Fig. 2A). Collagen type 1-positive connective tissues covering bone surface were thin in T1KO mice (*arrowheads*), and the immunoreactivity of Wnt3a and β-catenin in the mesenchymal tissues surrounding the OPN-positive neonatal bones was weak in T1KO mice (*arrows*) (Fig. 2B, Supplementary Fig. S4). The mRNA expression level of *β-catenin* was significantly lower in T1KO calvarial tissue and skin on P0 (Fig. 2C). Collagen fibers visualized by second harmonic generation (SHG) microscopy (shown in green) were irregular, thick and aggregated in the calvaria (Fig. 2D) and scalp (Fig. 2E) of T1KO mice on P0.

### Round skull shape in adult T1KO mice

**Figure 3:**
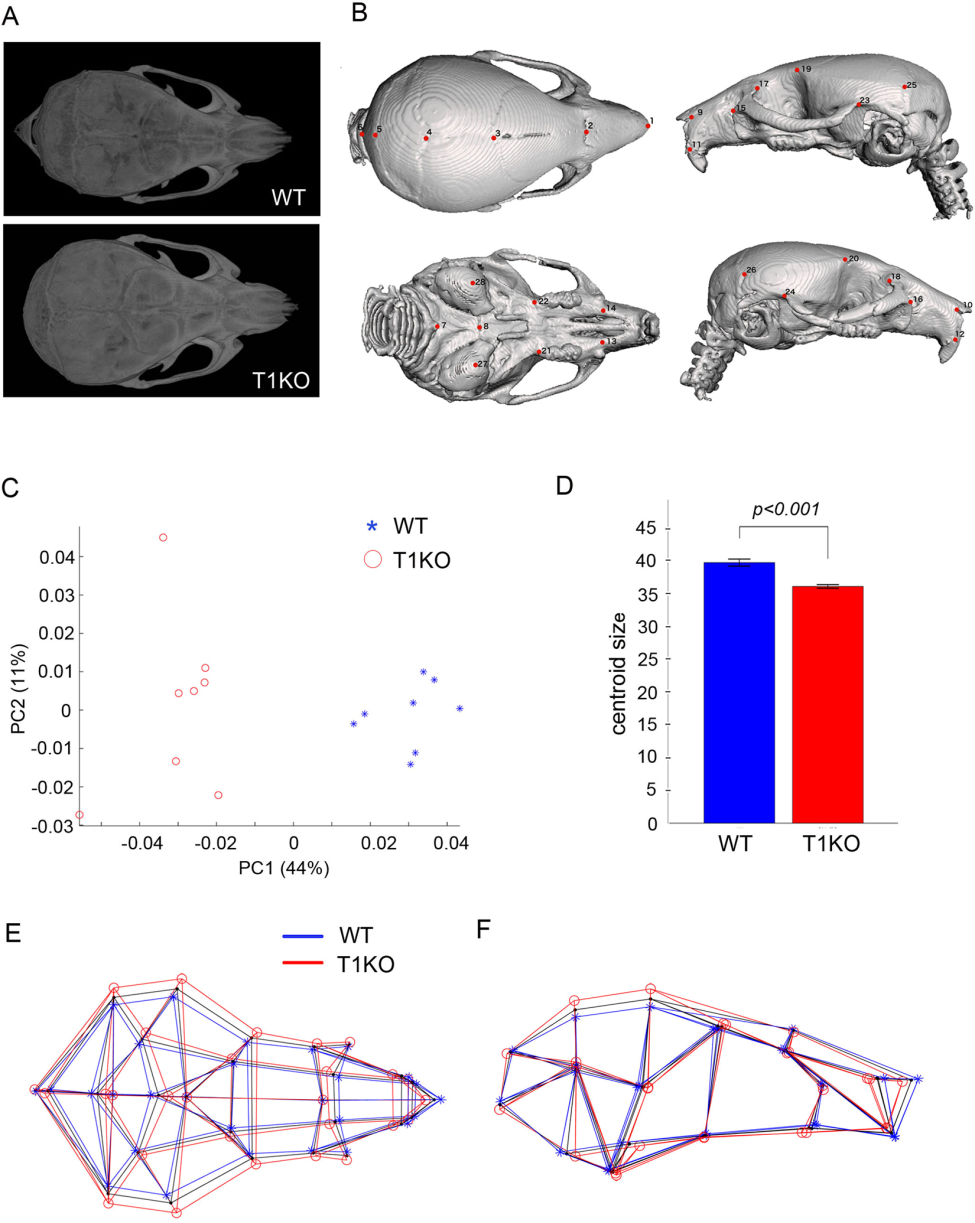
Analysis of craniofacial morphology in adult mice. The whole head in P28 was analyzed with a high resolution micro-CT apparatus. (**A**) Views from the parietal side. T1KO mice had round shaped skulls. (**B**) 3D landmarks used in this study from superior, inferior, left side, and right-side views (Supplementary Table S1). (**C**) Variation along PC 1 and 2 (UM1: open circles, UM2: asterisks). The total shape variation in the sample was 43.6% and 11.3%, indicating that T1KO and WT mice showed distinct craniofacial morphology from each other. (**D**) Bar graph showing centroid size as the measure of craniofacial size. (**E**,**F**) Representation of craniofacial shape differences between T1KO (*red wireframe*) and WT (*blue wireframe*), reconstructed from each negative and positive extreme along PC1 from the superior and the right-side views, respectively. The overall consensus configuration is shown by *black wireframe*. WT: n = 8, T1KO: n = 8, resampling test.

Around ninety one percent of T1KO mice developed without severe skeletal abnormality, however, they showed a rounded skull when compared with WT mice on P28 (Fig. 3A). To make the characteristic features of T1KO skull shape clearer, we analyzed the whole head using high-resolution micro CT and 3D coordination of 28 craniofacial landmarks (Fig. 3B, Supplementary Table S1). Graphing the first two PCs, which account for 43.6% and 11.3% of the total shape variation in the sample, indicates that T1KO and wild type mice show distinct craniofacial morphology (Fig. 3C). Differences between them along PC1 reflect the relative face length and the calvarial proportion. Compared with WT mice, T1KO mice demonstrated shorter faces, higher and broader calvaria and contraction of cranial bases (Fig. 3E,F). The shape difference between them is well supported by resampling (*p* < 0.001). Furthermore, we detected a significant difference in cranial size between T1KO and WT (*p* < 0.001; Fig. 3D).

### Deformation of cartilage tissue in the synchondrosis

We also checked endochondral ossification in the craniofacial region. The cranial base synchondrosis was deformed in T1KO mice on P28 (Supplementary Fig. S5), and the deformation of synchondrosis spheno-occipitalis in T1KO skull started on or before P7 (Supplementary Fig. S6). The disarrangement of chondrocytes which were immunopositive for aggrecan and collagen II were observed in T1KO catilage (Supplementary Fig. S6A,B), and immunopositive intensities of Wnt3a and β-catenin in the subchondral bone of synchondrosis spheno-occipitalis were weak in T1KO mice than in WT mice (Supplementary Fig. S6A,C).

### Severe developmental failure in the T1KO embryonic mice

T1KO mice could survive during embryonic days, but the T1KO pups rarely died in early embryonic days at E14.5 (Supplementary Table S2A). Although *CSGalNAcT1* (T1)*-* or *CSGalNAcT2* (T2)*-* null mice were viable and fertile, T1 and T2 double knockout mice were inviable due to completely lack of CS^33^ (Supplementary Table S2A). The average litter size at birth and growth rates until the weaning period was small in case of mating among T1(−/−) mice (Supplementary Table S2B). Approximately 5% of the surviving T1KO embryos showed severe developmental defects of their craniofacial regions such as eye defects, cranial deformation and vascular malformation on E18.5 (n = 2 in total 39 embryos) (Supplementary Fig. S7). Histological examination revealed that these T1KO embryos showed anaplasia of the nasal cartilage with nasal septum deficiency (*arrowhead*) and cleft palate (*arrows*) (Supplementary Fig. S7B). Furthermore, 4.2% of T1KO pups showed facial cleft including cleft lip (n = 2 in total 48 pups) (Supplementary Fig. S7C). To elucidate the cause of these severe abnormal features among the T1KO pups, we analyzed the CS amounts of neonatal pups. The T1KO mouse with severe abnormal phenotypes (T1KO (mal+)) had significantly low amounts of CS chains when compared with its littermate WT and T1KO mouse without severe abnormality (T1KO (mal−)) (n = 3 for each) (Supplementary Fig. S7D).

### Severe malformation of skull with malocclusion in adult T1KO mice

**Figure 4:**
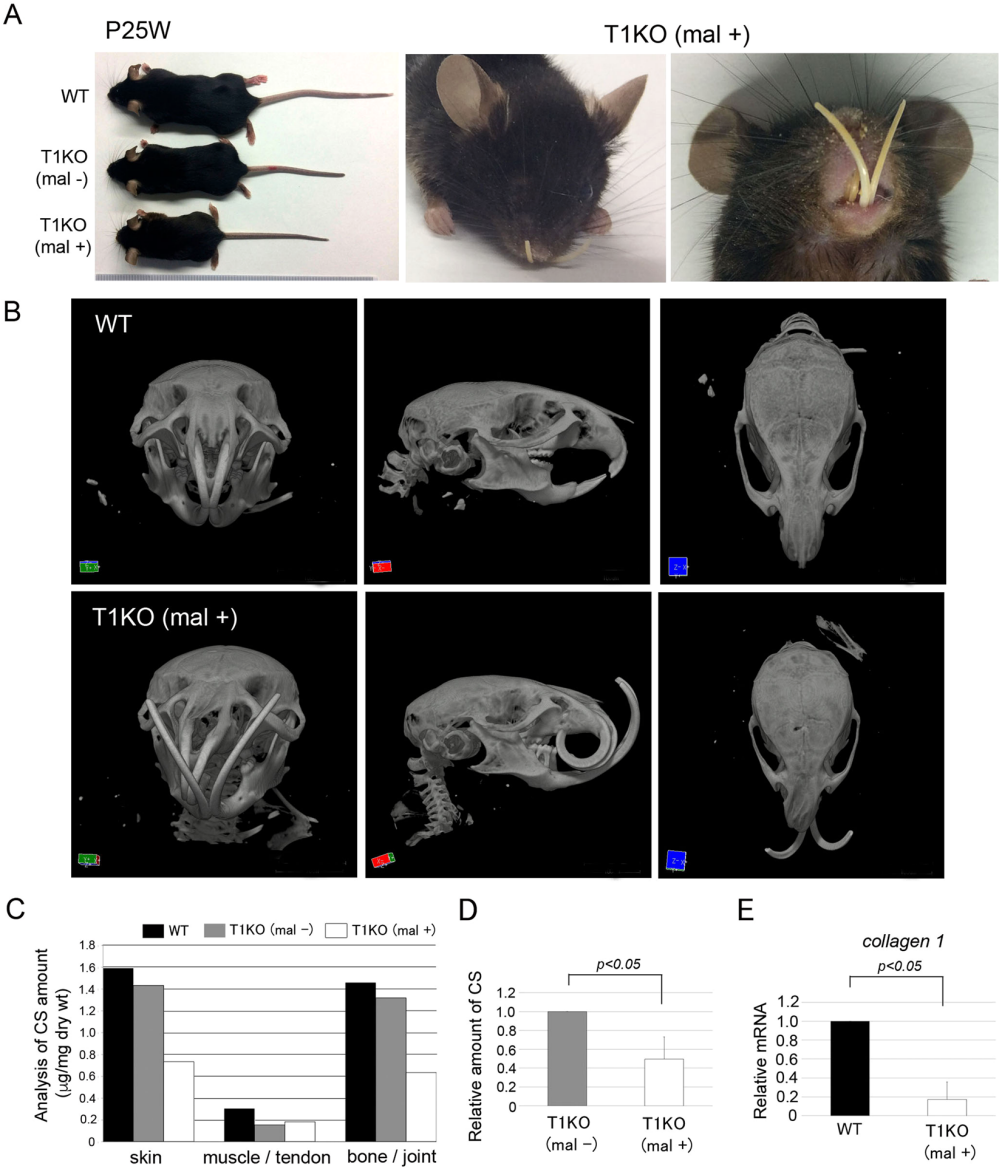
Deformation of skull with malocclusion in adult T1KO mice. Analysis of WT and T1KO mice with or without malocclusion in P25W. Micro CT analysis (**B**) and quantification of CS chains (**C**,**D**). T1KO with malocclusion (T1KO (mal+)) revealed the small body size, malocclusion with angle class III and eye defect (**A**). Micro CT photographs demonstrated the curved frontonasal regions, dysraphism of cranial suture, bone thinning and severe periodontitis in T1KO (mal+) mice (**B**). The amount of CS in T1KO (mal+) was remarkably lower in all tissues than that of WT and of T1KO (mal−) mice (**C**). The total amount of CS chains was significantly lower in T1KO mice with severe abnormalities such as malocclusion, skeletal dysplasia and facial cleft, compared with a littermate T1KO (mal−) (**D**). The mRNA expression of *collagen type 1* was significantly downregulated in T1KO (mal+) tail (**E**). n = 3, Student’s *t-*test.

**Figure 5:**
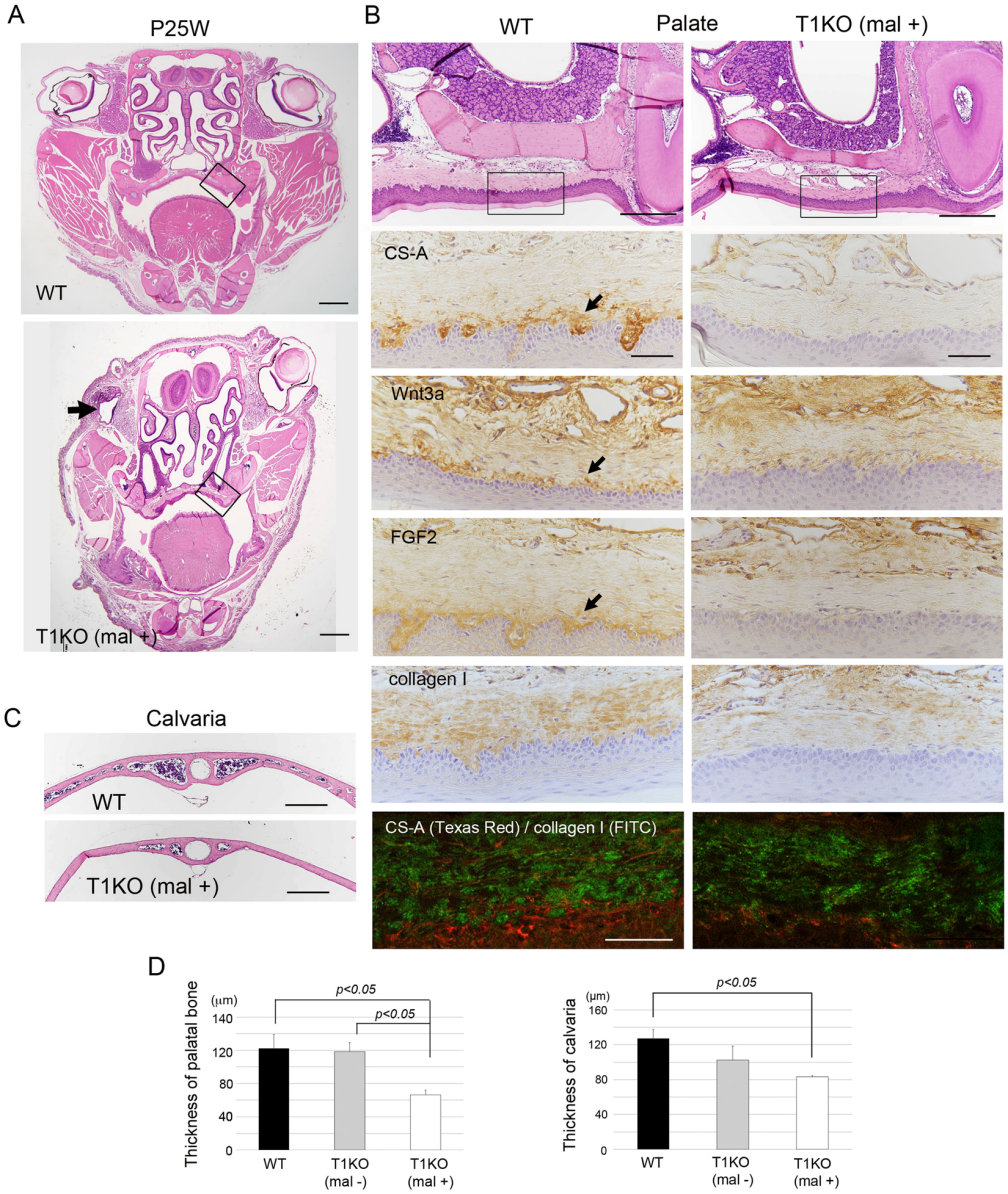
Histological analysis of adult T1KO mice with severe abnormal phenotype. Histological analysis of craniofacial region of P25W mice (see also Fig. 4). HE (**A**–**C**) and immunoperoxidase staining for CS-A, Wnt3a, FGF2 and collagen type 1, counterstained with hematoxylin (**B**). Double immunofluorescent staining of CS-A (Texs Red) and collagen type 1 (FITC) (**B**). T1KO (mal+) mice showed asymmetrical aspect and the unilateral eyeball was missing (*arrow*; **A**). In the mesenchymal tissue of palatal mucosa, the immunopositivities of Wnt3a and FGF2 were diminished with CS in T1KO (mal+) mice (*arrows*), and the immunostaining intensity of collagen type 1 in the lamina propria were weak in the T1KO (mal+) palate by both bright and fluorescence imaging (**B**). The palatal process of maxilla and calvaria were significantly thinner in T1KO (mal+) mice than in WT and T1KO (mal−) mice (**C,D**). (Palate: n = 4, calvaria: n = 3), ANOVA adjusted by Fisher LSD. Scale bars, 1 mm (**A**); 250 μm (**B**, *upper*); 50 μm (**B**, *lower*); 40 μm (**C**).

The T1KO pups without cleft palate and lip could survive after birth; however, 8.1% of them showed severe craniofacial malformation after 4 weeks of age (n = 10 in total 124 mice). These deformed mice exhibited small body size, malocclusion with angle class III and abnormal eyes (Fig. 4A). Micro CT images revealed a curved frontonasal region, dysraphism of cranial suture, bone thinning of cranium and mandible and severe periodontitis in T1KO mice (Fig. 4B). These phenotypes such as small body size, a curved frontonasal region, dysraphism of cranial suture and hypoplasia of bone were already observed in 4 week-old T1KO mice with malocclusion (Supplementary Fig. S8). They were similar to those of the osteogenesis imperfecta in mice^34,35^_._ To explore the relationship between the symptoms of skeletal dysplasia and the T1KO condition, the CS abundance and compositions of CS disaccharides were compared among the WT, T1KO without malformation (T1KO (mal−)) and with malformation (T1KO (mal+)) shown in Fig. 4A. In skin and bone tissues, the total amount of CS in T1KO (mal+) was remarkably lower than WT and T1KO (−) mice (Fig. 4C), a tendency similar to Supplementary Fig. S7D. Disaccharide composition of CS in each tissue is shown in Supplementary Table S3. We found that the remarkable reduction of CS abundance in T1KO mice was strongly related to severe abnormal phenotypes such as malocclusion, skeletal dysplasia (Fig. 4D). In addition, the mRNA expression of *collagen type 1* was significantly downregulated in T1KO (mal+) tail tissues (Fig. 4E). Histological analysis of craniofacial regions was performed using WT, T1KO (mal−) and T1KO (mal+) mice. In the frontal section, T1KO (mal+) mice showed asymmetrical aspects, and the unilateral eyeball was missing (Fig. 5A, indicated by *arrow*). The skeletal bones, such as the palatal process of maxilla and calvaria, were significantly thinner in T1KO (mal+) mice than those of WT and T1KO (mal−) mice (Fig. 5B–D). Next, we examined the mesenchymal tissue of palatal mucosa. The immunopositivities of Wnt3a and FGF2 in the lamina propria of palate were diminished with CS in T1KO (mal+) mice (*arrows*), and the immunostaining intensity of collagen type 1 in the lamina propria were weak in the T1KO (mal+) palate by both bright and fluorescence imaging (Fig. 5B).

### Downregulation of collagen type 1 and Wnt3a by T1 gene knockdown *in vitro* and *in vivo*

**Figure 6:**
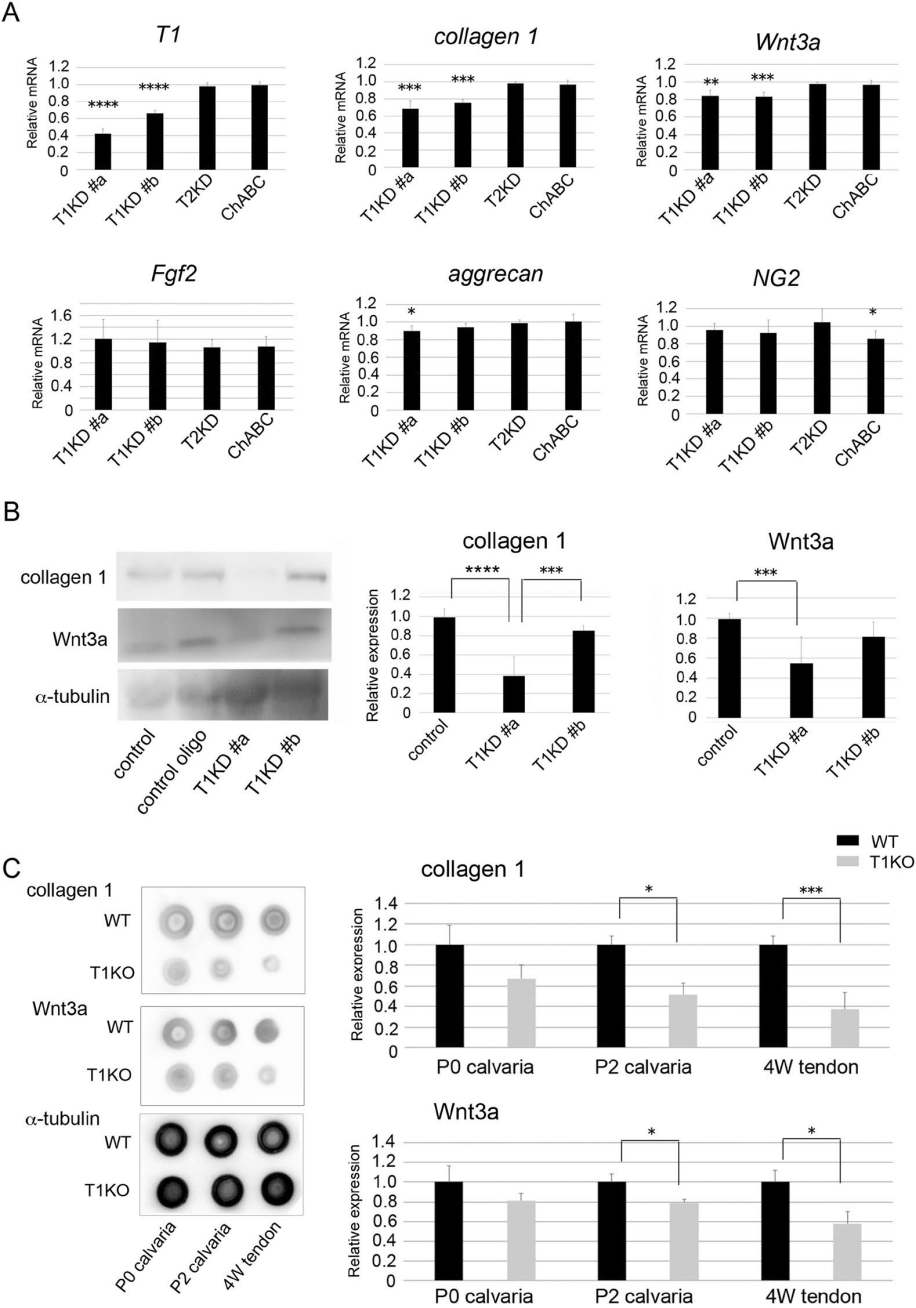
T1-targeted siRNA experiments in MC3T3-E1 cells and dot-blot analysis of T1KO tissues. (**A**) Quantitative real-time PCR analysis of siRNA experiments after 48 h in MC3T3-E1 cells. (**B**) Western-blot analysis of siRNA experiments after 72 h in MC3T3-E1 cells. The full-length blots are shown in Supplementary Fig. S9B. (**C**) Dot-blot analysis for proteins of WT and T1KO mice tissues. (**A**) The average expression of each gene in WT mice was defined as 1.0, and gene levels were compared with that of WT mice. mRNA expressions of *collagen type 1*, *Wnt3a and aggrecan* were downregulated by T1 gene knockdown, although T2 knockdown (T2KD) nor chondroitinase ABC (ChABC) treatment did not affect of them. The protein levels of collagen type 1 and Wnt3a after 72 h were significantly decreased by T1 gene knockdown by T1KD #a in MC3T3-E1 cells (**B**). T1KD #b, T2KD and ChABC did not suppress the protein expression of collagen type 1 and Wnt3a (**B**, Supplementary Fig. S9A). In P2 calvaria and 4W tendon tissues of T1KO mice, the protein amounts of collagen type 1 and Wnt3a were significantly reduced compared with that of WT mice (**C**). The average protein amount of each molecule in WT mice was defined as 1.0, and protein levels were compared with that of WT mice. *p < *0*.*05*, **p < *0*.*01*, ***p < *0*.*005*, ****p < *0*.*0001*, n = 5, Student’s *t-*test.

We found that mRNA expression and protein deposition of collagen type 1 and Wnt3a were reduced in several tissues in T1KO mice (Figs 1B,G, 2B, 4E and 5B). To clarify the effects of T1 gene knockout on collagen type 1 and CS-related molecule expression, we performed a T1-targeted siRNA experiment using two different sequences (T1KD #a and T1KD #b) and mouse MC3T3-E1 osteoblast-lineage cell lines. We have previously examined the efficiency of T1 gene knockdown of T1KD #a and T1KD #b, and confirmed that T1KD #a reduced *T1* mRNA expression more than T1KD #b^33^. As expected, the mRNA expression levels of *collagen type 1*, *Wnt3a* and *aggrecan* were significantly downregulated upon T1 gene knockdown after 48 h. Neither knockdown of T2, the isoform of T1, nor treatment with bacterial chondroitinase ABC (ChABC), an exogenous enzyme degrading CS, affected gene expression level of them (Fig. 6A). We also quantified the protein expression levels of these molecules after 72 h by western-blot analysis. The protein levels of collagen type 1 and Wnt3a were significantly decreased by T1 gene knockdown by T1KD #a in MC3T3-E1 cells (Fig. 6B), although T1KD #b construct did not significantly suppress them. It suggests that remarkable reduction of T1 gene by T1KD #a affects protein downregulation of them. The levels of FGF2 and β-catenin were not reduced by T1 gene knockdown (data not shown), and T2KD and ChABC did not suppress the protein expression of T1, collagen type 1 and Wnt3a (Supplementary Fig. S9A). The full-length blots are shown in Supplementary Fig. S9B. Next, to quantify the change of protein expression level by T1 gene knockdown *in vivo*, we performed dot-blot analysis using tissue samples of P0 calvaria, P2 calvaria and P4W tendon tissues in WT and T1KO mice. The average protein amount of each molecule in WT mice was defined as 1.0, and protein levels were compared with that of WT mice. The loading control protein of α-tubulin was stable among samples (Fig. 6C). The protein amounts of collagen type 1 and Wnt3a were significantly reduced in P2 calvaria and 4W tendon tissues of T1KO mice (Fig. 6C), similar to the immunohistochemical results and real time PCR data *in vivo* shown in Figs 1B,G, 2B, 4E and 5B.

### Ehlers-Danlos syndrome (EDS) -like phenotype in T1KO mice

**Figure 7:**
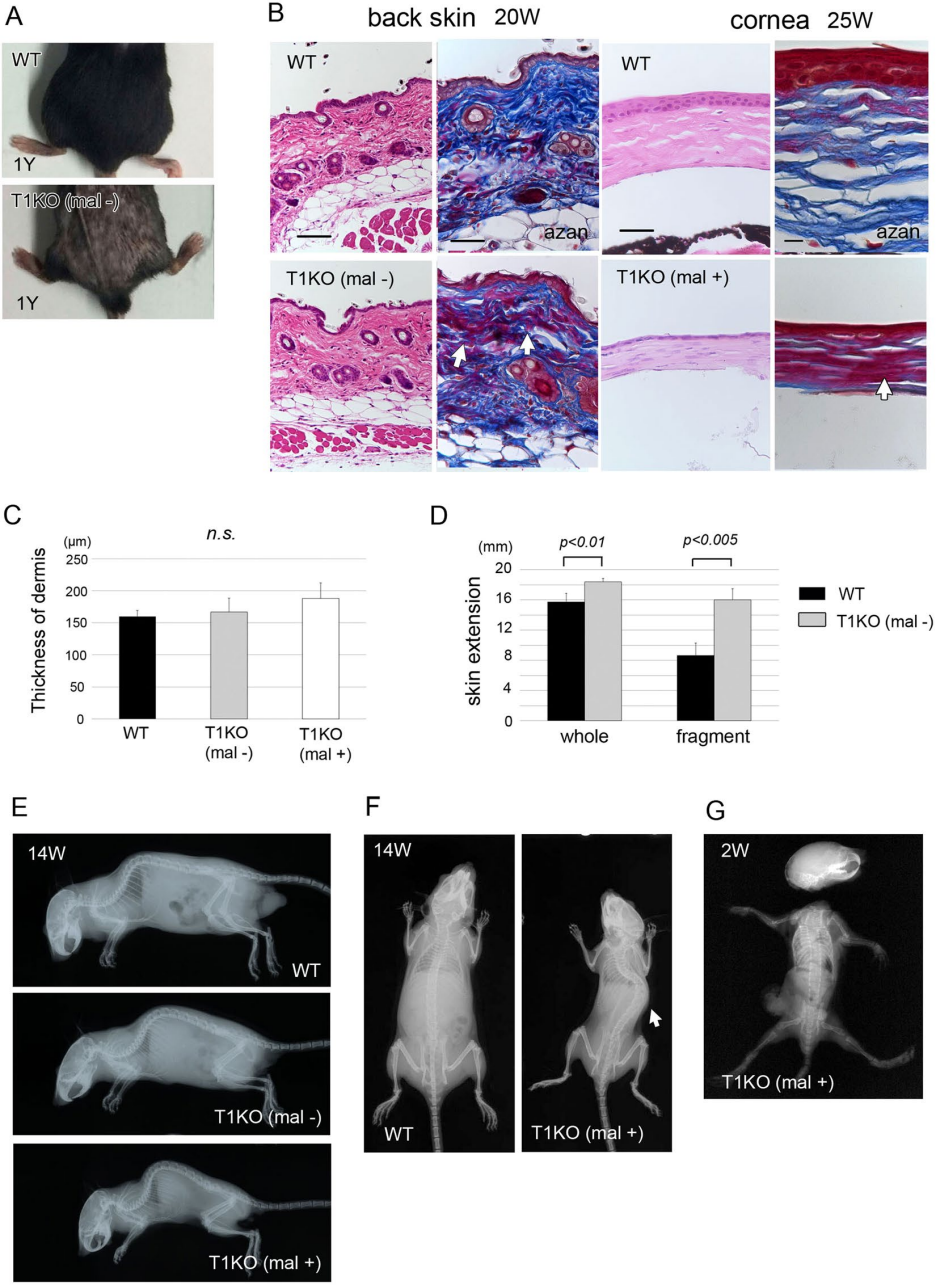
EDS-like phenotype in adult T1KO mice. (**A**) Gross appearance of back skin, (**B**) Histological analysis using HE and azan staining, (**E**–**G**) Soft X-ray analysis. Some T1KO aged mice with or without malformation showed atrophic skin with focal alopecia at 1Y (**A**). The collagen fibers of the dermis and cornea in T1KO mice were disordered and aggregated, as stained in red by azocarmine G (*arrows*) (**B**). The thickness of dermis of the back skin was not different among WT, T1KO (−) and T1KO (+) mice (**C**). Two different tension tests, using whole mice and skin fragments of back skin, were performed (Supplementary Fig. S10). Skin extension was significantly higher in T1KO (−) mice (whole: 18.375 ± 0.479 mm, fragment: 16.0 ± 1.472 mm, n = 5) than that of WT mice (whole: 15.75 ± 1.090 mm, fragment: 8.667 ± 1.607 mm, n = 4). T1KO mice having malocclusion showed severe scoliosis (**E**,**F**) and joint laxity (**G**). Student’s *t-*test (**C**,**D**). Scale bars, 100 μm (**B**).

Some of T1KO aged mice with or without severe malformation showed atrophic skin with focal alopecia at one year of age (Fig. 7A). The collagen fibers of the dermis and cornea in T1KO were disordered and aggregated as stained in red by azocarmine G at P20W and P25W (*arrows*) (Fig. 7B). The thickness of dermis of the back skin was not different between WT and T1KO mice with or without malformation (Fig. 7C). During routine handling of the mice, we noticed that the back skin of T1KO mice was hyperextensible when compared with WT mice. To confirm skin extensibility, we performed two different tension tests using whole mice and the skin fragments, as shown in Supplementary Fig. S10. The skin extension was significantly higher in T1KO (−) mice (whole: 18.375 ± 0.479 mm, fragment: 16.0 ± 1.472 mm) than in WT mice (whole: 15.75 ± 1.090 mm, fragment: 8.667 ± 1.607 mm) (Fig. 7D). In addition to these skin phenotypes, T1KO mice with malocclusion had symptoms of skeletal dysplasia with severe scoliosis (Fig. 7E,F) and joint laxity (Fig. 7G). These characteristic skeletal and skin features such as severe scoliosis, joint laxity and skin hyperextensibility are similar to the symptom of Ehlers-Danlos syndrome in humans^36,37^.

## Discussion

In the present study, we showed that a lack of the T1 gene caused disorder of intramembranous ossification, as well as cartilage formation in the cranium, resulting in the deformation of the craniofacial skeleton (Figs 1–5, Supplementary Figs S5–S8). This is the first report to demonstrate that CS is closely associated with the collagen fiber organization, and the marked reduction of CS induces severe craniofacial abnormality and skeletal dysplasia in T1KO mice, akin to the connective tissue disorder Ehlers-Danlos syndrome.

CSPG is a major component of cartilaginous tissue, and CS polymers are known to play a crucial role in endochondral ossification^28,38^. In this study, we found that T1KO mice showed a growth delay in intramembranous ossification of the skull at the neonatal stage, and the bone wall thinned in the craniofacial regions of adult mice. In the normal primary ossification of cranium, CS chains accumulated in condensed mesenchymal tissues of osteogenic front (Supplementary Fig. S1), colocalized with growth factors and signaling molecules. Therefore, it is reasonable that the remarkable reduction of CS chains induced the hypoplasia of intramembranous ossification as well as cartilage formation, resulting in skeletal dysplasia.

During craniofacial morphogenesis, some signaling molecules such as hedgehog, FGFs and Wnts are known to regulate skull shape^17,18,20^. We have shown that adult T1KO mice have shorter faces, higher and broader calvaria and skull abnormalities, similar to those reported in FGFR2 or FGFR3 mutant mice^39,40^. Because CS chains interact with FGF2 and Wnt3a, it is predicted that the localization of them may be affected in T1KO mice^11^. As expected, the immunostaining intensity of Wnt3a in the palate, calvaria and cartilage of cranial base were diminished in T1KO mice and the amount of Wnt3a protein reduced in some tissues of T1KO mice, although mRNA expression and protein level of FGF2 were not changed. Wnt3a regulates palate morphogenesis, and mutations in this gene cause cleft lip and palate in humans and mice^41,42^. Around 5% of T1KO pups had facial abnormalities showing cleft lip and palate. Thus, one of the pathogenesis of severe facial abnormalities in T1KO may be due to disorganized connective tissues caused by reduction of CS and CS-binding signaling molecules involved in ECM formation.

**Figure 8:**
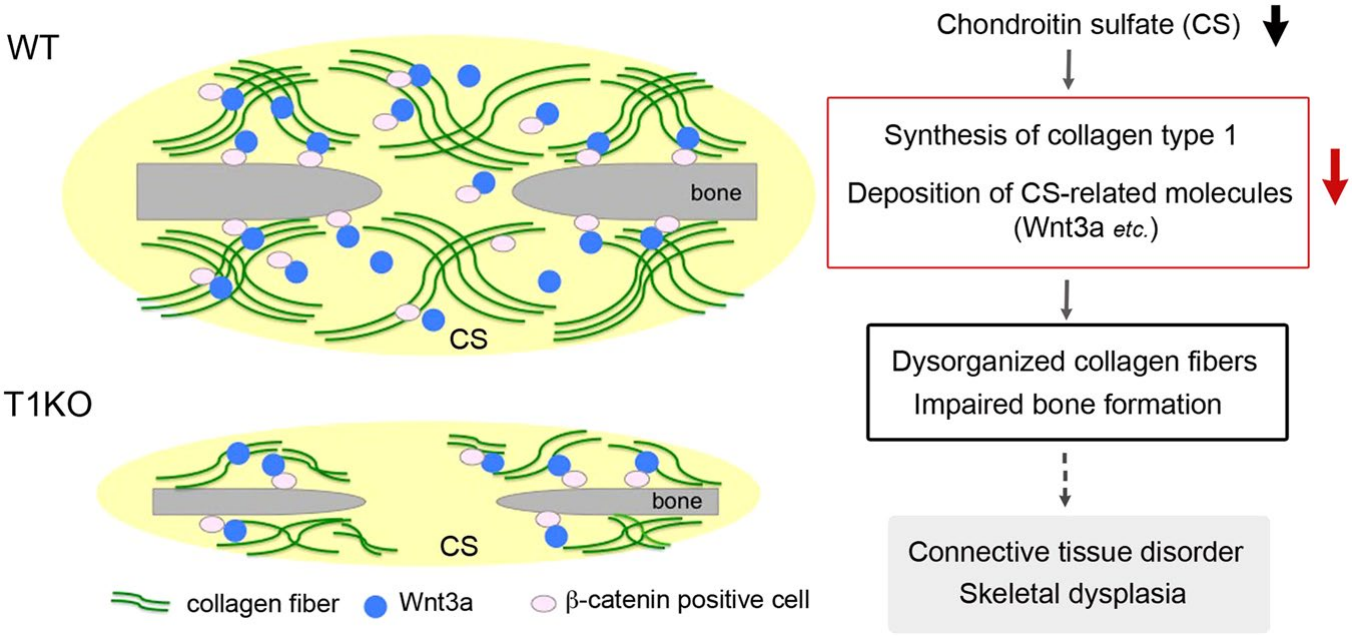
A schematic model for the mechanisms on hypoplasic intramembranous ossification in T1KO mice. Summary of the results concerning intramembranous ossification in T1KO mice. The reduction of CS in ECM suppresses the biosynthesis of collagen type 1 and deposition of CS-binding molecules such as Wnt3a. It may disturb the organization of collagen fibers (Fig. 2D,E), similar to the collagen-proteoglycan interaction in the collagen fibril architecture of the corneal stroma^46^. In addition, the lower expression levels of Wnt3a and β-catenin in the mesenchymal tissues surrounding the neonatal bones may induce the hypoplasia of craniofacial bones in T1KO mice (Figs 1, 2) because the canonical Wnt/β-catenin signaling promotes osteoblast differentiation^47^. Finally, the marked reduction of CS may induce connective tissue disorder including skeletal dysplasia. See Discussion in the text.

The mRNA expression of *collagen type 1* was significantly reduced in palatal and calvarial mesenchymal tissues in T1KO mice and was also downregulated in MC3T3-E1 osteoblast-lineage cells by T1 gene knockdown. CS is known to be bound to collagen type 1 fibrils and modulate osteoblast adhesion to ECM^43,44^ and CSPG is thought to regulate the collagen fibril architecture in the corneal stroma of bovine and mice^45,46^. Therefore, we speculated that the reduction of CS in ECM in T1KO mice may disturb the organization of collagen fibrils and its turnover in a direct or indirect pathway, resulting in connective tissue disorder. Because the mRNA expression of both collagen type 1 and its degrading enzyme, MMP13 (collagenase 3), were significantly reduced in T1KO palatal tissue (Figs 1G,H). The molecular mechanism of downregulation of collagen type 1 by T1 gene knockdown has not known well, but we speculate that T1 gene may locate the upstream of collagen type 1 gene. Because supression of collagen type 1 by T1 knockdown was observed both *in vivo* and *in vitro* in the present study, and gene knockdown of collagen type 1 by siRNA did not suppress the expression of T1 (data not shown). Further analysis is needed to elucidate the molecular regulatory mechanism of it. In addition, the abundance of β-catenin mRNA and the staining intensity of β-catenin protein was lower in calvaria and skin on P0 in T1KO compared to WT (Figs 1B, 2B,C, Supplementary Figs S2, S4). As the mRNA expression and protein level of β-catenin was not downregulated by the T1 gene knockdown in MC3T3-E1 cells, it is speculated that the local β-catenin expression in the cranium was affected by Wnt3a expression, not direct effect by T1 gene knockdown. The canonical Wnt/β-catenin signaling promotes osteoblast differentiation, and Wnt/β-catenin activity is essential to bone development^47^. These findings suggest that disorganized collagen fibrils with reduction of collagen type 1 and the decrease of Wnt3a/β-catenin expression in the mesenchymal tissues surrounding the neonatal bone may induce the hypoplasia of craniofacial bones in T1KO mice (Fig. 8).

In this study, we were able to detect that T1KO adult mice with low amount of CS showed skeletal dysplasia including abnormal craniofacial growth, dental malocclusion and curvature of the spine. In addition to these skeletal symptoms, these mice have anophthalmia, severe periodontitis and joint laxity with skin hyperextensibility. These features are closely related to the human Ehlers-Danlos syndrome and its mouse pathological models^34,35,48^. There are several lines of additional evidence on which these conclusions are based. (1) Recently, human missense mutation of the gene encoding CSGalNAcT1 has been reported to result in skeletal dysplasia and joint laxity^49^. (2) The loss of dermatan sulfate (a glycosaminoglycan species related to CS), due to lack of the enzymes such as galactosyltransferase 1 (B4GalT7), galactosyltransferase II (B3GALT6), or CHST14/dermatan 4-O-sulfotransferase-1 (D4ST1), are reported to cause EDS-like connective tissue disorder^48,50^. Since these enzymes are also involved in the synthesis of CS chains, it is likely that CS reduction causes similar disorders. (3) The abnormality of collagen fibrillogenesis is one of the causes of osteogenesis imperfecta and EDS^51^ and it is reasonable that our T1KO mice showed the phenotype of connective tissue disorder via reduction of CS and other related ECM molecules, including collagen type 1. Taken together, our findings suggest that there is a critical point in CS abundance to disturb fibrogenesis and its homeostasis, causing connective tissue disorder. Further analysis of skeletal dysplasia in the mice deficient in the enzymes synthesizing glycosaminoglycans will lead to the elucidation of the EDS pathogenesis for unidentified patients.

## Materials and Methods

### Animals

All the animal experiments were conducted in compliance with the protocol which was reviewed by the Institutional Animal Care and Use Committee and approved by the President of Niigata University (Permit Number: #28 Niigata Univ. Res. 42-9), and by President of Aichi Medical University (Permit Number: #2017-85). *Csgalnact1*^*tm1*.*1Migar*^ (ID: 234356) knockout (T1KO) mice were derived from the C57BL/6 N strain, and were genotyped and maintained as described previously^28^. For mice with malocclusion, finely crushed pellets were fed to avoid malnutrition.

### Micro CT and soft X ray analyses

Heads were examined using micro-computed tomography (micro CT) (Elescan; Nittetsu Elex, Tokyo, Japan). For measurement of skull morphology, samples of 8 wild-type controls and 8 T1KO mice at postnatal 4 weeks (P4W) were scanned with the following parameters: 72 kV, 100 µA, and voxel resolution of 34 μm. The 3D mesh models of mice skulls were reconstructed using ImageJ software (NIH, USA). For morphometric analysis, 3D coordinates of craniofacial landmarks were collected (n = 28). Figure 3B and Supplementary Table S1 provide locations and brief anatomical descriptions. Craniofacial shape variation was analyzed using standard geometric morphometric methods^52^. The generalized Procrustes fitting decomposes form into size and shape, such that size is measured as centroid size, which is defined as the square root of the sum of squared distances from the centroid of the landmark configuration to each landmark, and the shape was quantified as the deviation of the landmark configuration of each specimen from the consensus of all samples. Shape variation was explored via principal component analysis (PCA). Graphing the first few PC scores is a convenient means to explore statistically relevant patterns of shape variation in the sample and can be used for visual inspection. Resampling tests were used to test the difference between mutant and wild-type mouse cranium in mean-shape and centroid size. All calculations were performed in MATLAB 8.1 (MathWorks). For soft X-ray analysis, samples were taken with a SOFRON SRO-M40 (Sofron Co. Ltd, Tokyo, Japan). The exposure was at 30 kV and 4 mA and lasted for 20 s.

### Tissue preparation and immunohistochemistry

T1KO and WT littermates from embryonic days 15 (E15) to postnatal 25 weeks (P25W) were used for histological analysis. Mice were perfused with physiological saline followed by 4% paraformaldehyde in 0.1 M phosphate buffer (pH 7.4) under deep anesthesia. Following decalcification in Morse’s solution for 3–6 days at 4 °C, the samples were processed for embedding in paraffin, and 4 µm-thick serial sections were stained with hematoxylin-eosin (HE), azocarmine and aniline blue (Azan) and alcian blue, and processed for immunohistochemistry using the antibodies listed in Supplementary Table S4. The Envision+/HRP system (Dako, Glostrup, Denmark) and the avidin-biotin peroxidase complex (ABC) (Vectastain ABC kit; Vector Laboratories, Burlingame, CA, USA) method were used for immunohistochemical staining. For aggrecan, FGF2 and versican, sections were pretreated with 3 mg/ml bovine testicular hyaluronidase (type I-S, 440 U/mg; Sigma Chemical Co., St Louis, MO) in PBS at 37 °C for 30 min. For collagen type 1, β-catenin and osteopontin, sections were autoclaved in citric acid buffer (pH 6.0) at 121 °C for 5 minutes. For visualization of reaction products, sections were treated with 3,3′-diaminobenzidine (DAB) (Dohjindo Laboratories, Kumamoto, Japan) in the presence of 0.005% hydrogen peroxide and counterstained with hematoxylin. For control experiments, the primary antibodies were replaced with preimmune rabbit IgG or mouse IgG_._ To compare the staining intensities between WT and T1KO sections, we performed all tissue preparation procedures and immunostaining steps at the same time, including the time for final color development with DAB solution. For double immunofluorescent staining, Texas Red-conjugated anti-mouse IgG + IgM (Rockland, Gilbertsville, PA, USA), FITC- conjugated anti-rabbit IgG (Vector Laboratories Inc.) or FITC-conjugated streptavidin (Vector Laboratories Inc.) were applied. The sections were examined with a confocal laser-scanning microscope (FV300, Olympus, Tokyo, Japan).

### Quantitative real-time PCR analysis

Palatal and calvarial tissues were dissected from 3 WT and 3 T1KO pups at P0 under stereomicroscopy as shown in Fig. 1F. Total RNA was isolated from the dissected tissues using the Trizol system (Nippon Gene Co., Ltd., Tokyo, Japan). First-strand cDNA was synthesized with the SuperScript First-Strand Synthesis System (Invitrogen, Carlsbad, CA, USA). Quantitative analysis of gene expression was performed by qRT-PCR using SYBR1 Premix Ex Taq II (Takara, Otsu, Japan; RR820A) and oligonucleotide primers specific for the target sequences (Supplementary Table S5) on a Thermal Cycler Dice (Takara). Amplification conditions were as follows: 30 s at 95 °C; 40 cycles of 95 °C for 5 s and 60 °C for 30 s; dissociation for 15 s at 95 °C; and 30 s at 60 °C. Gene expression levels were calculated relative to the levels of β-actin mRNA using the comparative Ct (2−ΔΔCt) method.

### RNAi in cell culture

Murine MC3T3-E1 pre-osteoblast cell lines were obtained from JCRB cell bank (National Institute of Biomedical Innovation). Stealth Select RNAi (Invitrogen) was used to knockdown *Csgalnact1 and Csgalnact2* expression. In some cultures, ChABC (2 mU; Sigma Chemical Co.) was added 12 h after transfection. RNA sequences of the siRNAs used in these experiments are listed in Supplementary Table S6. The scrambled sequences of the negative-control siRNAs had 4 or 5 nucleotides that differed from the corresponding nucleotides in the targeted siRNAs. Total RNA was extracted from cells 48 h after transfection, and performed real-time qPCR analysis as described previously^33^. The nucleotide sequences of real-time qPCR primers and ZEN double-quenched probes are listed in Supplementary Table S6.

### Western blot and dot blot analyses

For western blot analysis, MC3T3-E1 cells were collected 72 h after siRNA treatment, and subjected to SDS/PAGE (10% gels), followed by immunoblot analysis using antibodies as listed in Supplementary Table S4.

For dot blot analysis, calvarial bone tissues with scalp from WT and T1KO pups at P0 and P2, and the tendon tissue of lower limb from WT and T1KO mice at P4W were dissected under stereomicroscopy (n = 3 for each). Ten pg of protein extracts were blotted to the nitrocellulose membrane, and probed with the antibodies as listed in Supplementary Table S4. Antigens were detected using ECL Plus Western Blotting Detection Reagents (GE Healthcare). Signals were acquired using the Light-Capture II cooled CCD camera system (ATTO Corp., Tokyo, Japan) and quantified via a densitometric assay using CS Analyzer 3.0 software (ATTO).

### Quantification analysis of CS chain

CS chain analysis was conducted by enzymatic treatment and HPLC-based quantification as described previously^28,33,53^. Samples were prepared from the limb or tail in WT and T1KO mice from postnatal day 0 to 25 weeks. GAGs were extracted from each sample by incubating the samples in a protease solution (0.01 mg of actinase E, 10 mM of CaCl_2_, 50 mM of Tris–HCl (pH 8.0)) at 55 °C for 2 days. After the addition of trichloroacetate, each extract was centrifuged at 15,000 g for 20 min. Each partially purified CSPG fraction was digested with ChABC (5 mIU of ChABC in 60 mM CH3COONa, 50 mM Tris–HCl (pH 8.0)). GAGs in each digest were derivatized using 2-aminobenzamide; these mixtures were analyzed.

### Analysis of collagen fibers

Label-free multi-photon imaging was used to study mouse skin and skull. To perform SHG image acquisition, we utilized an upright multi-photon excitation microscope (A1R-MP, Nikon, Inc.). The microscope was equipped with a water immersion objective lens (CFI75 Apo 25 × W MP, NA:1.1, Nikon, Inc.) and a Ti:Sapphire laser oscillator system (MaiTai eHP, Spectra-Physics, Inc.) with no additional optical modules for generating polarized light. The images were acquired as z-stack image sequences with a step size of 5 µm ranging from the deepest portions to the surface of the tissue. All SHG images were acquired at an excitation wavelength of 960 nm. To acquire the SHG signal and autofluorescence from tissue, we divided signals at 495 nm by employing a dichroic mirror^54,55^. The field of view of the acquired images was 0.5 mm × 0.5 mm, and the resolution was 1024 × 1024 pixels, i.e., the pixel size was 0.5 µm.

### Skin Tension Test

Two types of skin tension tests were performed using WT mice (n = 4, average body weight: 29.2 g) and T1KO mice (n = 5, average body weight: 25.1 g) in P18W as shown in Supplementary Fig. S10. First, we pulled the dorsal skin by the forceps until the abdomen was detached from the floor (Supplementary Fig. S10A). In the second experiment, a skin fragment of 5 mm × 25–28 mm in size was prepared from back skin and pulled with a weight of 50 g (Supplementary Fig. S10B). Differences in skin tensile properties between the WT and T1KO mice were analyzed by Student’s *t-*test.

### Statistics

All data are presented as the means and standard errors of each group. StatPlus for Mac (AnalystSoft Inc., Vancouver, BC, Canada) was used to perform statistical analyses. Student’s *t*-test assuming equal variances was used for single comparisons and one-way analysis of variance (ANOVA) adjusted by Fisher LSD was used for multiple comparisons.

To analyze growth rate during embryonic stage, we performed Chi-square test.

## Electronic supplementary material

Supplementary data
