# Supplementary material for: Craniofacial abnormality with skeletal dysplasia in mice lacking chondroitin sulfate N-acetylgalactosaminyltransferase-1
Source: Sci Rep. 2018 Nov 20;8:17134. doi: 10.1038/s41598-018-35412-5 (PMC6244165; doi:10.1038/s41598-018-35412-5)
Supplement: Supplementary file 1 — Supplementary data [file 41598_2018_35412_MOESM1_ESM.pdf]

Craniofacial abnormality with skeletal dysplasia in mice lacking chondroitin sulfate *N*-acetylgalactosaminyltransferase-1

Hiroko Ida-Yonemochi, Wataru Morita, Nobuo Sugiura, Ryosuke Kawakami, Yuki Morioka, Yuka Takeuchi, Toshiya Sato, Shunichi Shibata, Hideto Watanabe, Takeshi Imamura, Michihiro Igarashi, Hayato Ohshima and Kosei Takeuchi

Supplementary Information

Supplementary Table 1: Landmarks for Micro CT analysis

Supplementary Table 2: Growing condition of CSGalNAcT1 mutant pups

Supplementary Table 3: Compositions of CS disaccharides

Supplementary Table 4: Antibodies used in the immunodetection

Supplementary Table 5: The oligonucleotide primers used for Real-time PCR analysis

Supplementary Table 6: List of the siRNAs

Supplementary Figure 1: Localization of CS in normal craniofacial development in mice

Supplementary Figure 2: Double immunostaining of bone and CS-related molecules in P0 palate.

Supplementary Figure 3: Quantitative real-time PCR analysis from the palate

Supplementary Figure 4: Double immunostaining of bone and CS-related molecules in P0 calvaria.

Supplementary Figure 5: The cranial base synchondrosis on P0 and P28

Supplementary Figure 6: The synchondrosis spheno-occipitalis on P7

Supplementary Figure 7: Severe developmental failures in T1KO embryos and neonatal pups

Supplementary Figure 8: Morphology of T1KO mice with malocclusion in P4W

Supplementary Figure 9: Appendix data of Figure 6.

Supplementary Figure 10: Schematic models of the skin tension tests

Supplementary Table 1. Landmarks for Micro CT analysis

Number	Side	Descriptions
1	M	nasale
2	M	nasion
3	M	bregma
4	M	intersection of parietal and interparietal bone
5	M	intersection of interparietal and occipital bones at the midline
6	M	Opistion
7	M	Basion
8	M	Sphenobasion
9	L	anterior-most point at intersection of premaxillae and nasal bones
10	R	anterior-most point at intersection of premaxillae and nasal bones
11	L	center of alveolar ridge over maxillary incisor
12	R	center of alveolar ridge over maxillary incisor
13	L	most inferior point on premaxilla-maxilla suture
14	R	most inferior point on premaxilla-maxilla suture
15	L	anterior notch on frontal process lateral to infraorbital fissure
16	R	anterior notch on frontal process lateral to infraorbital fissure
17	L	intersection of frontal process of maxilla with frontal and lacrimal bones
18	R	intersection of frontal process of maxilla with frontal and lacrimal bones
19	L	frontal-squasmosal intersection at temporal crest
20	R	frontal -squasmosal intersection at temporal crest
21	L	intersection of maxilla and palatine on inferior alveolar ridge
22	R	intersection of maxilla and palatine on inferior alveolar ridge
23	L	joining of squasmosal body to zygomatic process of squasmosal
24	R	joining of squasmosal body to zygomatic process of squasmosal
25	L	intersection of parietal, temporal and occipital bones
26	R	intersection of parietal, temporal and occipital bones
27	L	most inferior point on tympanic bulla
28	R	most inferior point on tympanic bulla

Supplementary Table 2. Growing condition of CSGalNAcT1 mutant pups

A. The viable ratio of mutant embryos.

Genotype	E12.5				E14.5			
	viable	inviable	χ2	*p*	viable	inviable	χ2	*p*
T1 (+/+)	50 (28.1%)	0			14 (26.9%)	0		
T1 (+/-)	86 (48.3%)	0	0.921	0.631	26 (50.0%)	0	0.154	0.926
T1 (-/-)	42 (23.6%)	0			10 (19.2%)	2		
Total	178 (100%)	0			50 (96.1%)	2 (3.9%)		
T2 (+/+)	29 (25.9%)	0			10 (23.8%)	0		
T2 (+/-)	52 (46.4%)	0	0.643	0.7251	21 (50.0%)	0	0.048	0.976
T2 (-/-)	31 (27.7%)	0			11 (26.2%)	0		
Total	112 (100%)	0			42 (100%)	0		
T1 (-/-):T2 (+/-) x T1 (-/-):T2 (+/-)								
T1 (-/-) : T2 (+/+)	28 (24.6%)	0			15 (23.8%)	0		
T1 (-/-) : T2 (+/-)	55 (48.2%)	0	27.675	0.000	28 (44.4%)	0	14.395	0.001
T1 (-/-) : T2 (-/-)	0 (0%)	31			0 (0%)	20		
Total	83 (72.8%)	31 (27.2%)			43 (68.2%)	20 (31.8%)		

(+/+) wild type mice: (+/-): heterozygous mice, (-/-): null mice

B. Growth rates until the weaning period.

Genotype	Average litter size (n)	Growth rate (%)	Total (n)
WT x WT	6.8	70.5	72
T1 (+/-) x T1 (+/-)	6.5	68.8	99
T1 (-/-) x T1 (-/-)	4.2	49.3	82
T2 (+/-) x T2 (+/-)	6.4	70.7	38
T2 (-/-) x T2 (-/-)	6.2	69.8	42
T1 (-/-):T2 (+/-) x T1 (-/-):T2 (+/-)	4.4	42.9	50

Supplementary Table 3. Compositions of CS disaccharides

(%)

	Type	0S	4S	6S	SE	SB	SD	total
Skin	WT	12.6	71.7	6.8	1.3	7.1	0.5	100
	T1KO (mal -)	15.7	68.3	5.5	1.2	9	0.3	100
	T1KO (mal +)	6.7	73.1	5.5	1.4	13	0.3	100
Muscle/ Tendon	WT	34.2	52.1	1	12.6	0.1	0	100
	T1KO (mal -)	41.9	48.8	1	0.5	7.8	0	100
	T1KO (mal +)	29.6	56.5	1.6	0.6	11.7	0	100
Bone/ Joint	WT	13.8	75.6	7.6	1.2	1.5	0.3	100
	T1KO (mal -)	5.9	85.5	2.5	4.2	1.8	0.1	100
	T1KO (mal +)	9.7	79.1	4	3.3	3.6	0.3	100

Supplementary Table 4. Antibodies used in the immunodetection

Antibody	Supplier	Species	Dilution for IHC	Dilution for WB/Dot blot
CS-A (2H6)	Seikagaku Co.	Mouse Monoclonal IgM	1:200	
Versican	Abcam	Rabbit Monoclonal	1:100	
Aggrecan	Abcam	Rabbit Polyclonal	1:200	1:500
Collagen type I	Abcam	Rabbit Polyclonal	1:250	1:1000
Collagen type II	Abcam	Rabbit Polyclonal	1:200	
FGF2	Santa Cruz Biotechnology	Rabbit Polyclonal	1:500	1:1000
Wnt3a	Bioss	Rabbit Polyclonal	1:500	1:1000
β-catenin	Santa Cruz Biotechnology	Rabbit Polyclonal	1:100	1:1000
Osteopontin	Santa Cruz Biotechnology	Mouse Monoclonal	1:100	
Ki67	Dako	Rat Monoclonal	1:100	
α-tubulin	MBL	Rabbit Polyclonal		1:1000
CSGalNacT1	Our group[28]	Rabbit Polyclonal		1:500

Supplementary Table 5. The oligonucleotide primers used for Real-time PCR analysis

Molecule	Primer sequence	Genebank accession number
β-actin		NM_007393
Forward	5'-TGGAATCCTGTGGCATCCATGAAAC-3'	
Reverse	5'-TAAAACGCAGCTCAGTAACAGTCCG-3'	
Collagen 1		NM_007742
Forward	5'-CACCCTCAAGAGCCTGAGTC-3'	
Reverse	5'-GTTCGGGCTGATGTACCAGT-3'	
Mmp13		NM_008607
Forward	5'-AAGTGTGACCCAGCCCTATC-3'	
Reverse	5'-GGGAAGTTCTGGCCAAAAGG-3'	
Wnt3a		NM_009522
Forward	5'-TTCTTACTTGAGGGCGGAGA-3'	
Reverse	5'-CTGTCGGGTCAAGAGAGGAG-3'	
Fgf2		NM_008006
Forward	5'-CCAACCGGTACCTTGCTATG-3'	
Reverse	5'-TATGGCCTTCTGTCCAGGTC-3'	
Cyclin D1		NM_007631
Forward	5'-GCGTACCCTGACACCAATCT-3'	
Reverse	5'-CACAACTTCTCGGCAGTCAA-3'	
Versican		BC096495
Forward	5'-GCTGTGGATGGTGTTGTGTT-3'	
Reverse	5'-CCCTTCCTTCCCCATCATGT-3'	
NG2		NM_139001
Forward	5'-CGTCAAGAGTCTCAACAGCG-3'	
Reverse	5'-GTGGCCACAAACGGGATATC-3'	
Aggrecan		NM_007424
Forward	5'-AGGTTGCTATGGTGACAAGG-3'	
Reverse	5'-TGGAAGGTGAATTTCTCTGGG-3'	
Csgalnact1		NM_172753
Forward	5'-GAAAGGGACTGGATGTTGGAG-3'	
Reverse	5'-AAATACCTTCTTCCCTGGCTG-3'	
β-catenin		NM_007614
Forward	5'- ATGGAGCCGGACAGAAAAGC -3'	
Reverse	5'- TGGGAGGTGTCAACATCTTCTT -3'	

Supplementary Table 6.　List of the siRNAs

siRNA target		Target sequence
Csgalnact1 (Chondroitin sulfate-N-acytyl –galactosaminyltransferase1)	Csgalnact1 T1-KD#a	GCAAUCAAAGGCUAUGAAUGA (2179-2199)
	Csgalnact1 T1-KD#b	GGUUCUGUUCAGUCAGUAUAA (1849-1869)
Csgalnact2 (Chondroitin sulfate-N-acytyl –galactosaminyltransferase2)	Csgalnact2 T2-KD#a (T2-KD)	GCAGAGCCAGUAUCUCCUUUG (3411-3431)
Csgalnact1 control sequence	Csgalnact1 T1-KD#a control	GCUAUCUAUGGCAUUGAUUGA
	Csgalnact1 T1-KD#b control	GGUACUGUACUGUCAGAAUAA

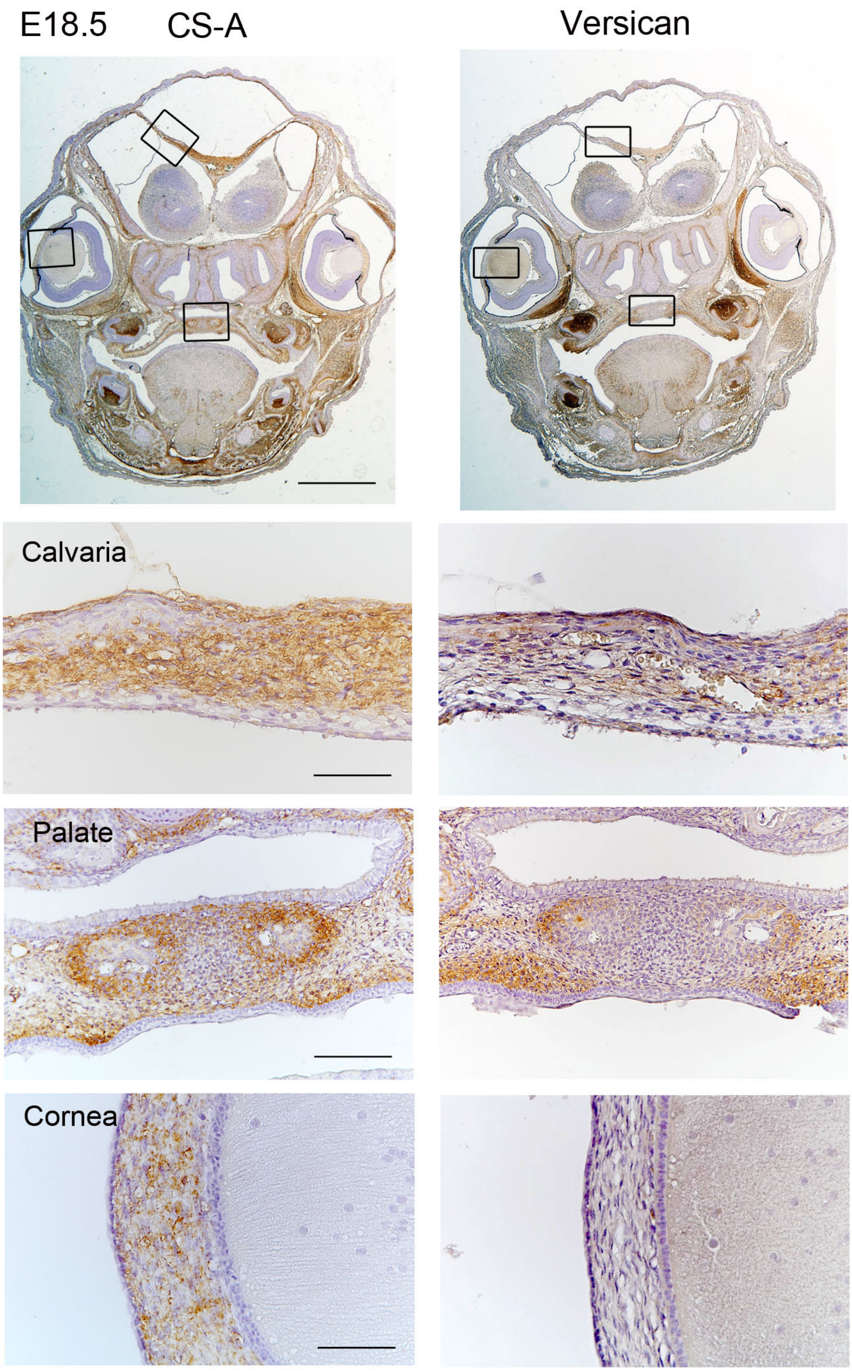

Supplementary Figure 1. Localization of CS in normal craniofacial development in mice.
The immunostaining of CS (CS-A antibody) and versican, using hematoxylin counterstain in WT mice on E18.5. CS was widely distributed in the facial regions, colocalized with versican at the embryonic stages. In magnified views of CS-A and versican, CS was immunolocalized in the mesenchymal cells surrounding newly formed bones in the calvaria, palate and cornea. Versican showed expression patterns similar to those of CS chain.
Scale bars, 1 mm (upper), 50 μm (lower).

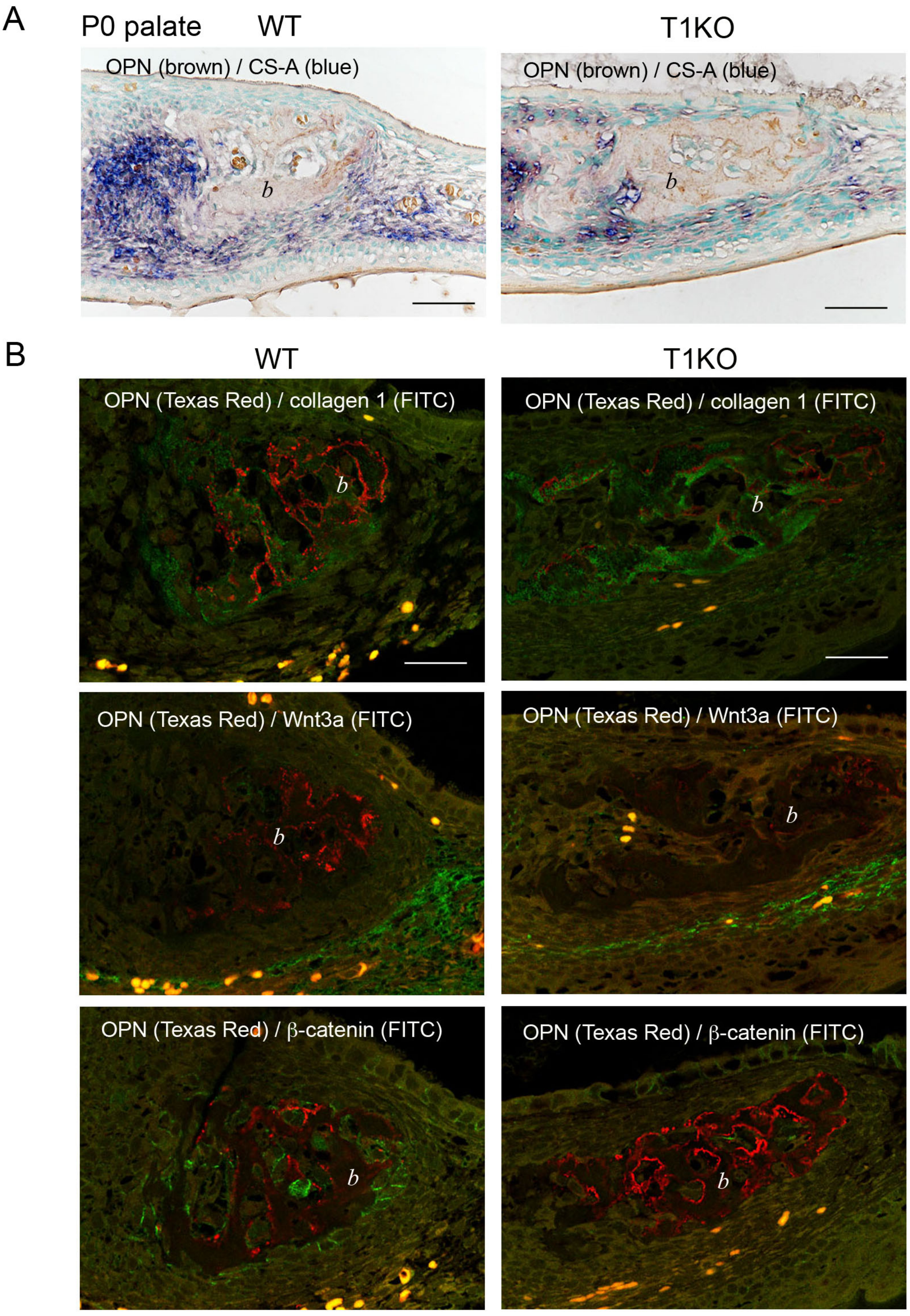

Supplementary Figure 2. Double immunostaining of bone and CS-related molecules in P0 palate. (A) Double immunoperoxidase staining of OPN (DAB, brown) and CS-A (ALP, blue). (B) Double immunofluorescent staining of OPN (Texas Red) and collagen 1 (FITC), Wnt3a (FITC) and β-catenin (FITC). OPN was strongly immunostained in the periphery of neonatal bones by immunofluorecent staining method. Collagen 1 was stained in the bone matrices framed by OPN-positive lines. CS-A, Wnt3a and β-catenin were widely distributed in the mesenchymal tissues surrounding bone in WT palate comparing with that of T1KO mice (A, B). Non-specific staining was observed in red cells (yellow). *b*: bone tissue. Scale bar, 50 μm (A), 33 μm (B).

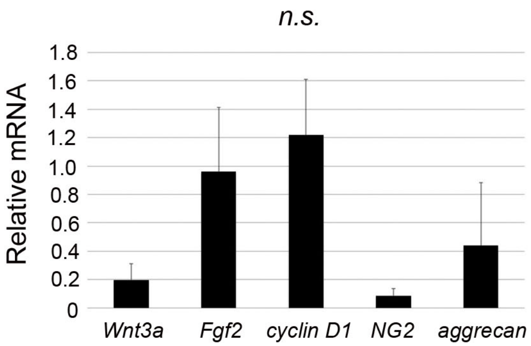

Supplementary Figure 3. Quantitative real-time PCR analysis from the palate.
Palatal tissue of WT and T1KO mice at P0. The average expression of each gene in WT mice was defined as 1.0, and gene levels were compared with that of WT mice. The mRNA expressions of *Wnt3a, Fgf2, cyclin D1, NG2* and *aggrecan* were not significantly different between WT and T1KO mice, although they tended to be low in the T1KO palate. n = 3, Student's t test.

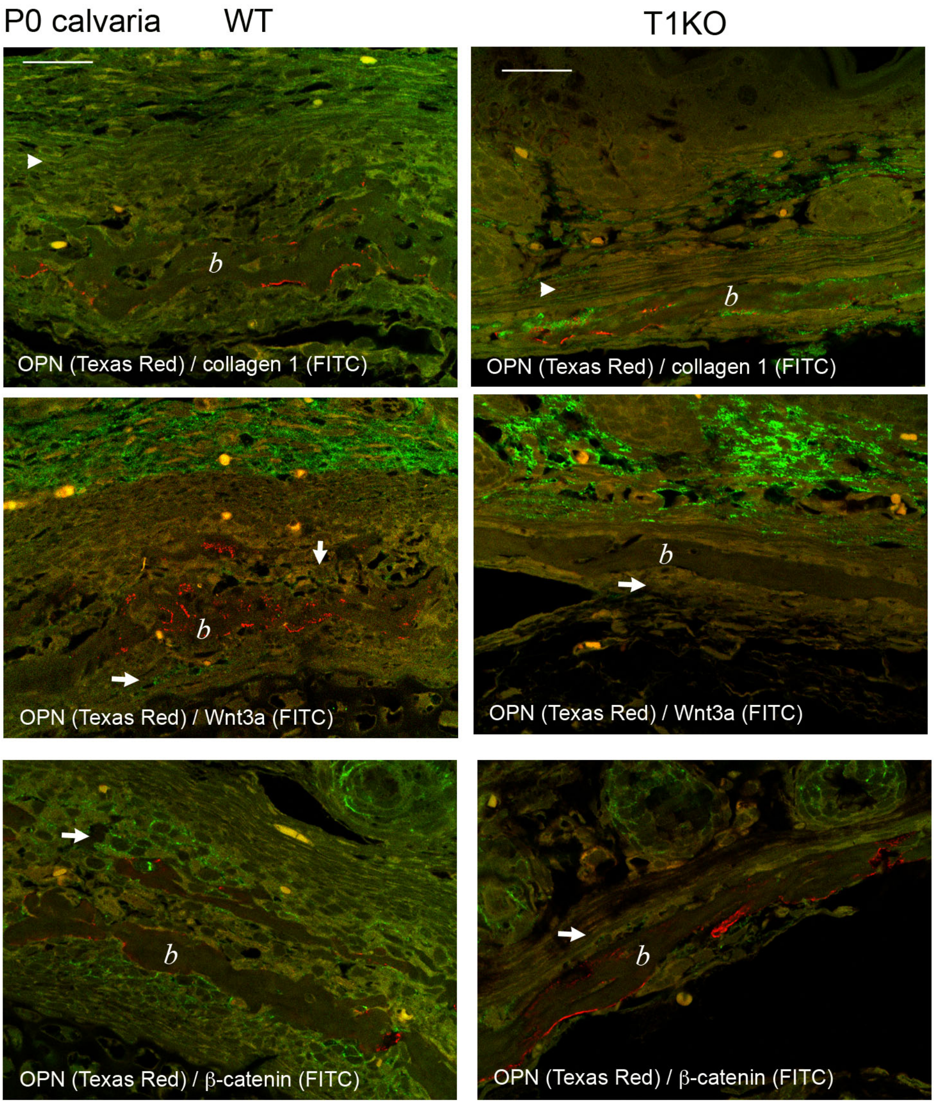

Supplementary Figure 4. Double immunostaining of bone and CS-related molecules in P0 calvaria. Double immunofluorescent staining of OPN (Texas Red) and collagen 1 (FITC), Wnt3a (FITC) and β-catenin (FITC). Collagen 1 was stained in the bone matrices framed by OPN-positive lines and connective tissues around bone (arrowheads). The immunoreactivity of Wnt3a and β-catenin in the mesenchymal tissues, surrounding the OPN-positive neonatal bones, was weak in T1KO mice (arrows). Non-specific staining was observed in red cells (yellow). *b*: bone tissue. Scale bar, 33 μm.

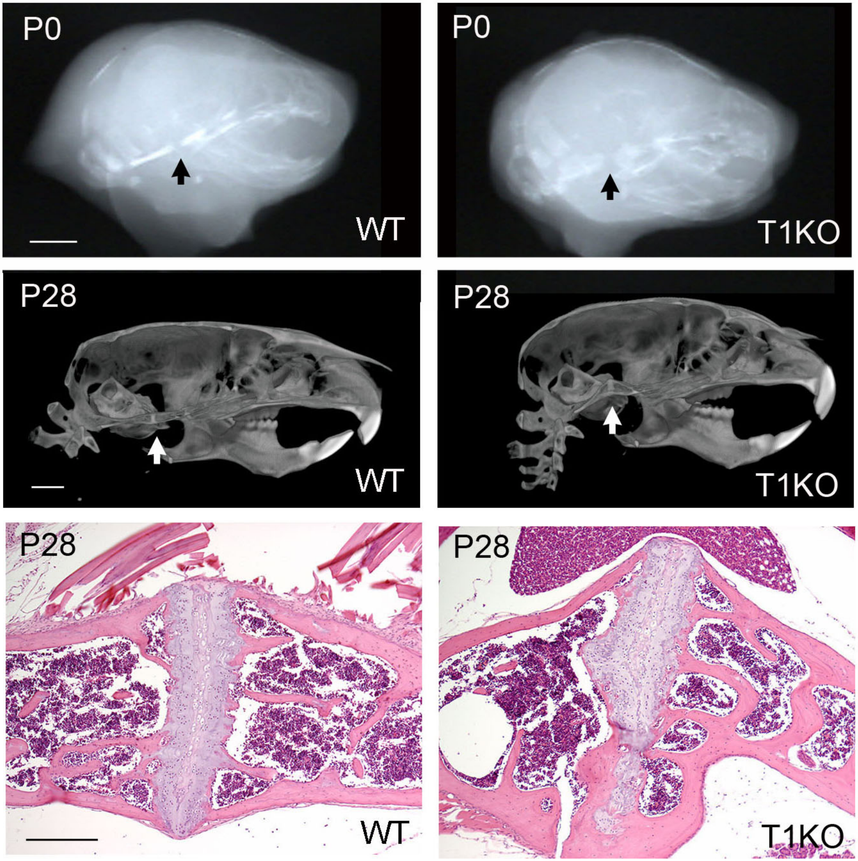

Supplementary Figure 5. The cranial base synchondrosis on P0 and P28.
The heads were evaluated by soft X ray (P0), micro CT images and histological sections of HE stain on P28. Ossification of cranial base was delayed in T1KO mice on P0, and the cranial base synchondrosis was deformed (arrow) in T1KO mice at P28. Scale bars, 2 mm (upper, middle) and 250 μm (lower).

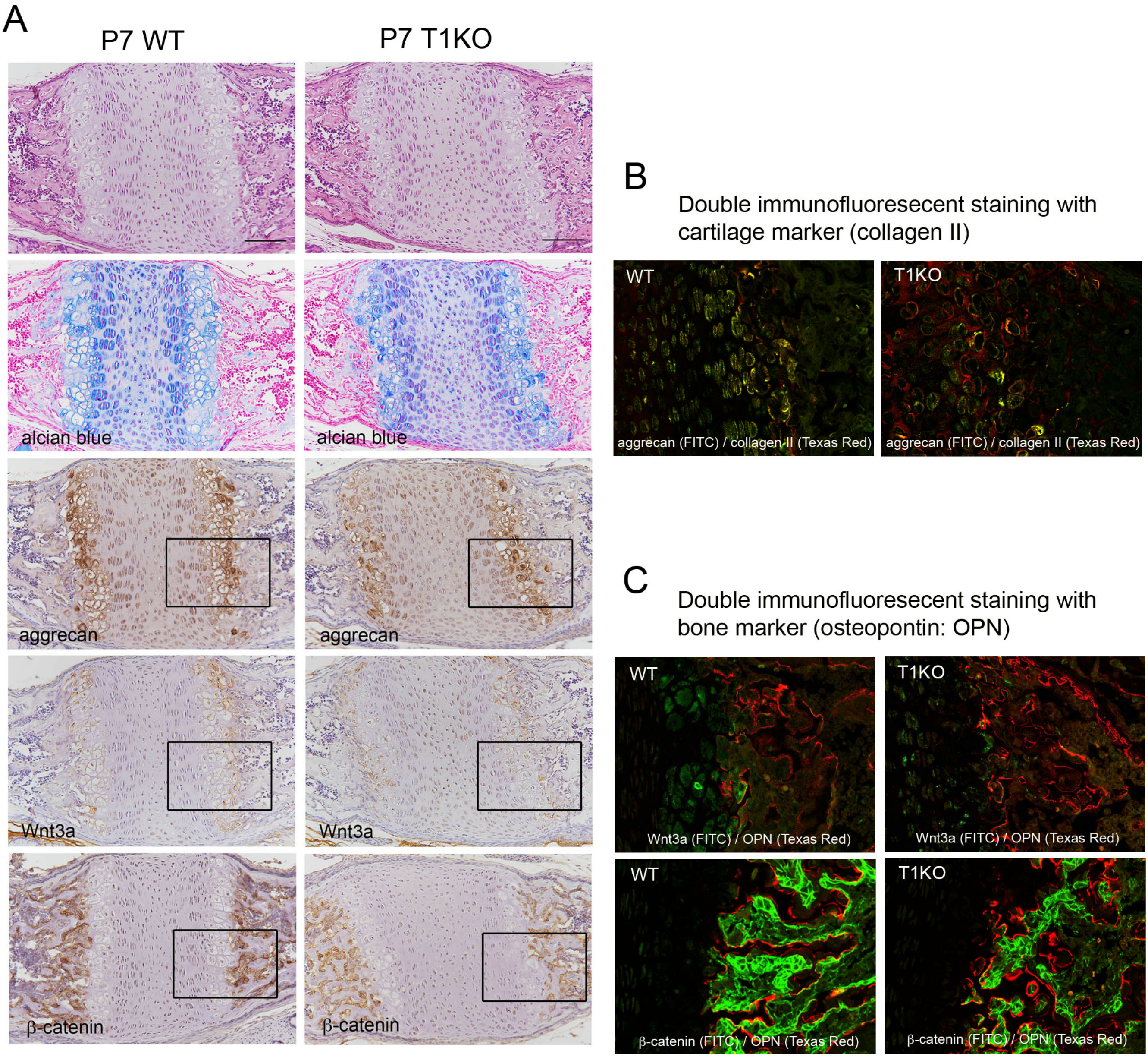

Supplementary Figure 6. The synchondrosis spheno-occipitalis on P7.
(A) HE stain, alcian blue stain and immunoperoxidase stain for aggrecan, Wnt3a and β-catenin counterstained with hematoxylin. (B, C) Double immunofluorescent staining with cartilage and bone markers. The disarrangement of chondrocytes and deformation of synchondrosis spheno-occipitalis were observed in T1KO mice on P7 (A, B). The immunoreactivity of Wnt3a and β-catenin was faint in the chondro-osseous junction of T1KO (A, C). Scale bar, 50 μm.

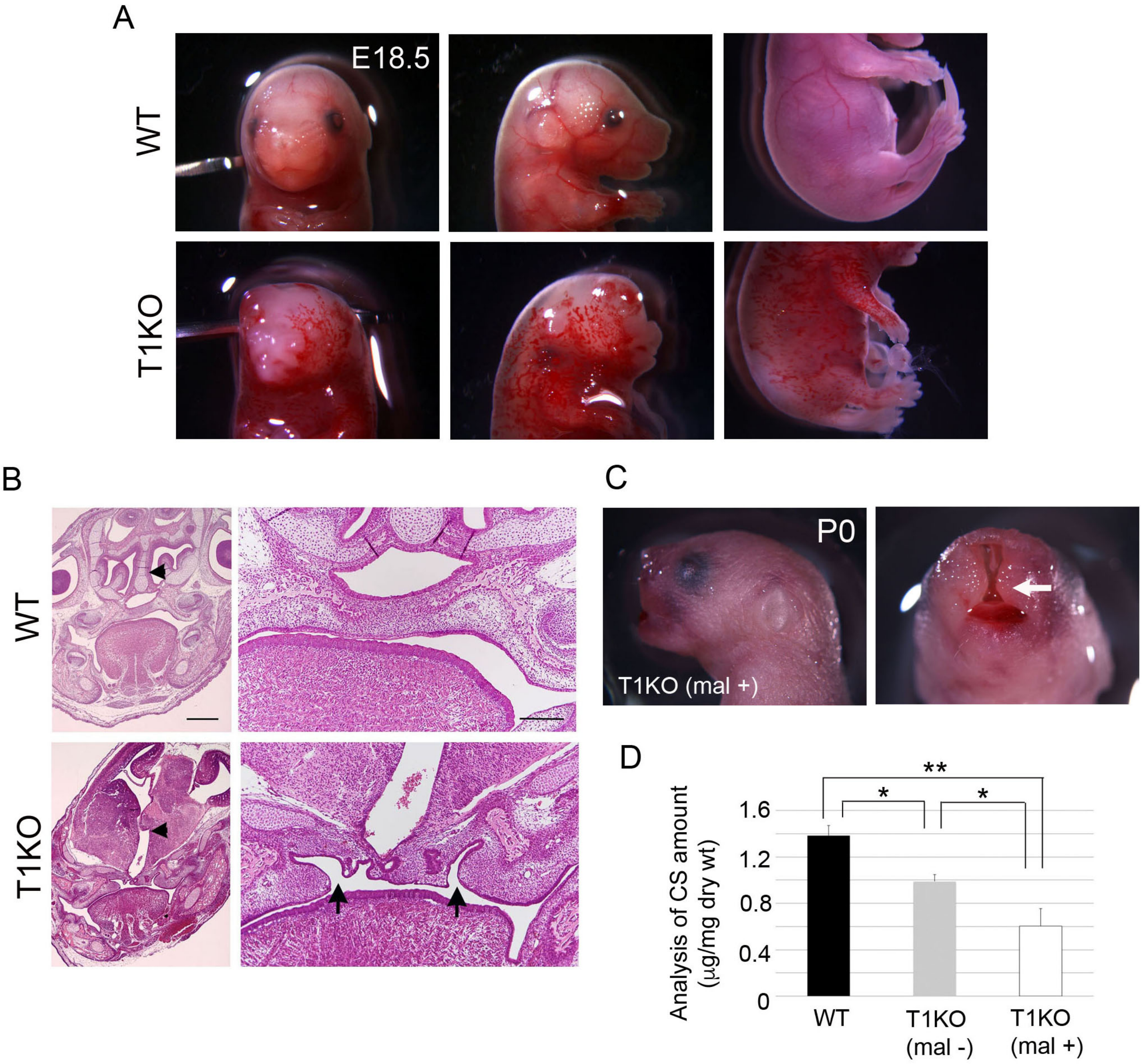

Supplementary Figure 7. Severe developmental failures in T1KO embryos and neonatal pups. T1KO embryos and neonatal pups with severe developmental defects. Stereoscopic microscopic views of E18.5 (A) and P0 (C) pups. (B) Histological analysis using HE staining. Eye defect, cranial deformation and vascular malformation were observed in T1KO on E18.5 (A). T1KO embryos showed the hypoplasia of the nasal cartilage, with nasal septum deficiency (arrowhead) and cleft palate (arrows) (B). Some of T1KO pups showed the facial cleft at birth, including the cleft lip (C). Remarkably low amount of CS chains was confirmed in T1KO mice with severe malformation (mal +) in P0 pups (D). n = 3 for each, ANOVA adjusted by Fisher LSD. *$p<0.01$, **$p<0.0001$. Scale bars, 500 μm (B, left); 250 μm (B, right).

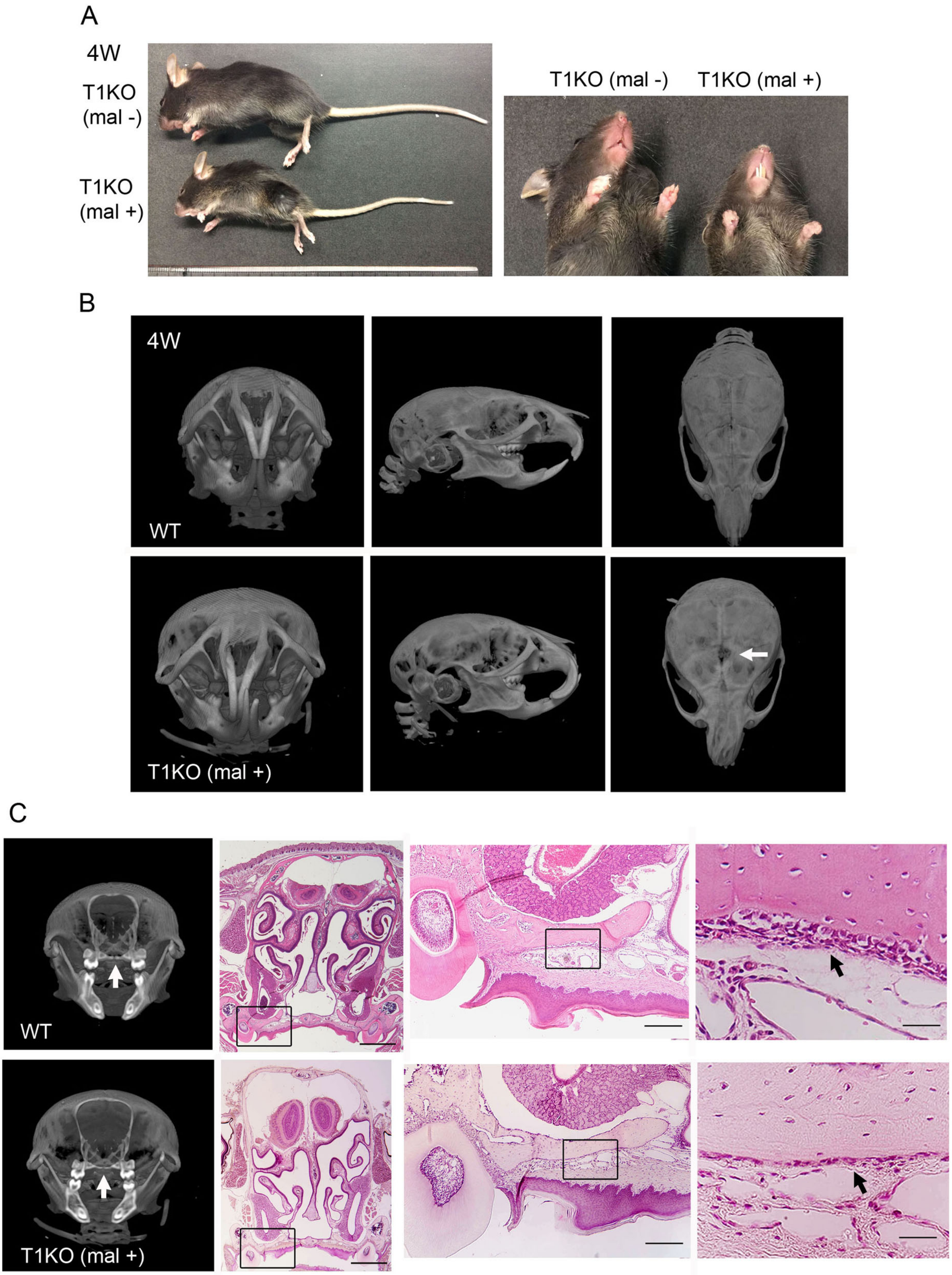

Supplementary Figure 8. Morphology of T1KO mice with malocclusion in P4W.
Analysis of WT and T1KO mice with malocclusion in P4W. Micro CT analysis (B, C) and histological analysis of craniofacial region (C). T1KO with malocclusion (T1KO (mal +)) revealed the small body size (A). Micro CT photographs demonstrated the curved frontonasal region and dysraphism of cranial suture (*arrow*) in T1KO (mal +) mice (B). T1KO (mal +) mice showed asymmetrical aspect of nasal cavity, and the palatal process of maxilla was thin with few osteoblast-lineage cells in T1KO (mal +) mice comparing with WT mice (*arrows*) (C). Scale bars, 1 mm (C, left), 250 μm (C, middle), 50 μm (C, right).

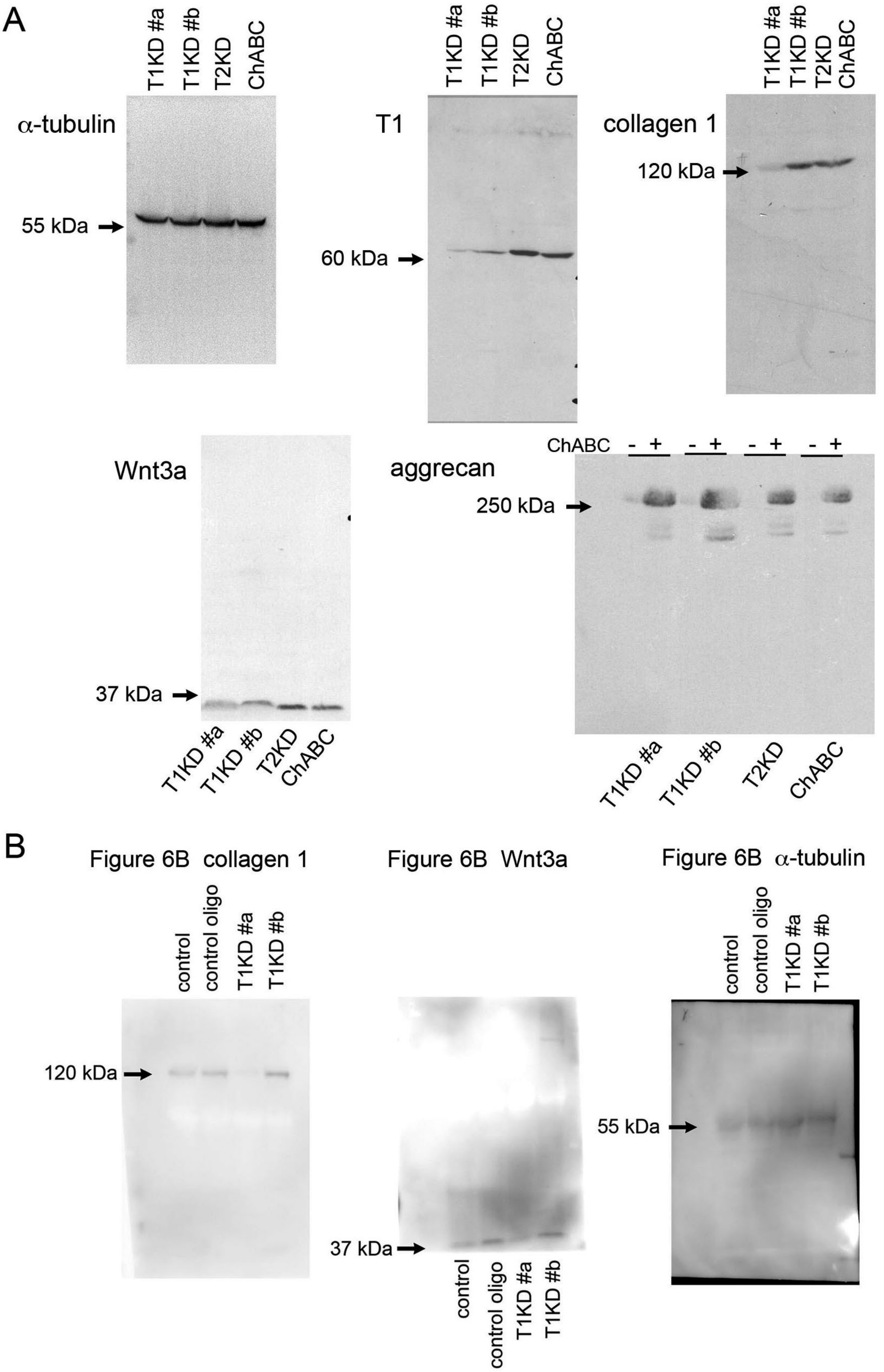

Supplementary Figure 9. Appendix data of Figure 6.
(A) Western-blot analysis of T1KD siRNA, T2KD siRNA and ChABC treatment after 72 h in MC3T3-E1 cells. T2KD and ChABC did not suppress the protein expression of T1, collagen 1 and Wnt3a. The antibody against aggrecan could detect aggrecan protein after ChABC treatment (+), and its expression was not affected by T1KD, T2KD and ChABC. Loading control, α-tubulin, was constant among samples. (B) Full-length blotting membranes of western blot analysis in Figure 6B.

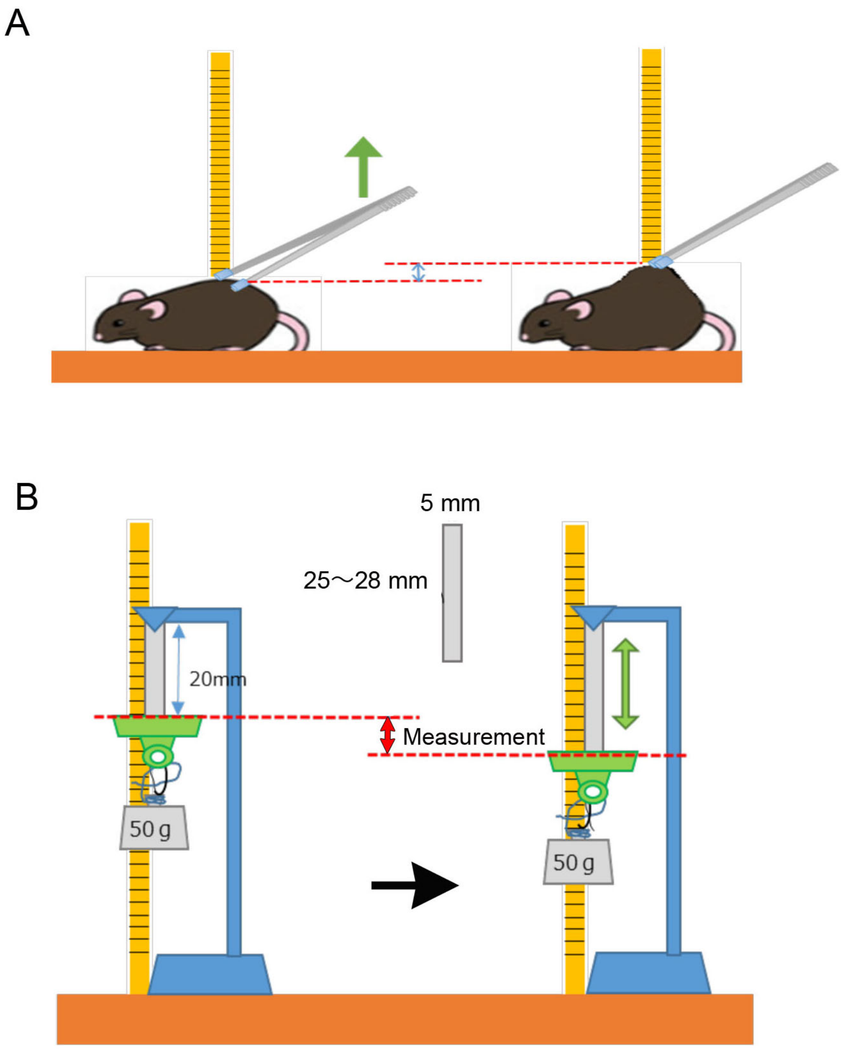

Supplementary Figure 10. Schematic models of the skin tension tests.
Two types of skin tension tests are shown. First, we pulled the dorsal skin of mice using the forceps until the abdomen was lifted from the floor (A). In the second experiment, a 5 mm x 25-28 mm skin fragment was prepared from back skin of both WT and T1KO mice and pulled by a weight of 50 g (B).
